# Layered Defect-Filling co-Assembled Carbazole-Based SAMs Deliver 20% Organic Solar Cells and 17% Mini-Modules

**DOI:** 10.1007/s40820-026-02325-2

**Published:** 2026-08-10

**Authors:** Jingnan Wu, Fengbo Sun, Leandro R. Franco, Qiaonan Chen, Xinxin Xia, Ruike Zhou, Zhuang Li, Mateus Bergami, Pablo Hermosilla Fernandez, C. Moyses Araujo, Biao Xiao, Xunchang Wang, Donghong Yu, Maojie Zhang, Renqiang Yang, Ergang Wang

**Affiliations:** 1 Department of Chemistry and Chemical Engineering, Chalmers University of Technology, 412 96 Göteborg, Sweden; 2 Key Laboratory of Special Functional Aggregated Materials (Shandong University), Ministry of Education, School of Chemistry & Chemical Engineering, National Engineering Research Center for Colloidal Materials, Shandong University, Jinan, 250100 People’s Republic of China; 3 Key Laboratory of Flexible Optoelectronic Materials and Technology (Ministry of Education), School of Optoelectronic Materials & Technology, Jianghan University, Wuhan, 430056 People’s Republic of China; 4 Department of Engineering and Physics, Karlstad University, 651 88 Karlstad, Sweden; 5 Materials Theory Division, Department of Physics and Astronomy, Uppsala University, 751 20 Uppsala, Sweden; 6 Department of Chemistry and Bioscience, Aalborg University, 9220 Aalborg, Denmark; 7 Sino-Danish Center for Education and Research, 8000 Aarhus, Denmark

**Keywords:** Organic solar cells, Interface engineering, Self-assembled monolayers, Trap passivation, Scalable photovoltaics

## Abstract

Self-assembled monolayers (SAMs) are widely used as hole-transport layers (HTLs) in organic solar cells (OSCs), yet conventional single-component SAMs often form quasi-monolayers with incomplete coverage and interfacial defects that become increasingly detrimental upon device scaling. Here, we develop a co-assembled multilayered SAM (coSAMu) strategy that combines two SAM molecules, 2PACz and 2Cl-4PACz, with distinct dipoles and steric configurations through blend casting and sequential casting. Photoelectron spectroscopy, X-ray analysis, and molecular simulations support a layered structure in which a chemisorbed, 2PACz-rich bottom layer primarily sets the indium tin oxide (ITO) work function, while a 2Cl-4PACz-rich upper layer fills interfacial voids, improves molecular packing, and passivates defects. Consistent with this picture, coSAMu promotes a more favorable vertical composition near the ITO surface and suppresses trap-assisted recombination, enabling more efficient charge extraction and collection. Consequently, a representative D18:L8-BO OSC incorporating the sequential-cast coSAMu HTL achieves a power conversion efficiency of 20.1% (0.042 cm^2^), outperforming pristine 2PACz. Importantly, when scaled to a 17.14 cm^2^ mini-module (six serially connected subcells), coSAMu delivers 17.0% efficiency versus 12.2% for the 2PACz control. This work demonstrates controlled multilayer co-assembly as an effective strategy for scalable OSC interface engineering that is broadly applicable to multiple donor–acceptor systems.

**Supplementary Information:**

The online version contains supplementary material available at 10.1007/s40820-026-02325-2.

## Highlights

- A sequential co-assembly of 2PACz and 2Cl-4PACz forms densely packed, electronically coupled SAM-based hole-transport layers.
- The bottom 2PACz-rich monolayer tunes the indium tin oxide work function, while the upper 2Cl-4PACz layer fills interfacial voids, enhances packing, improves coupling with donor polymers, and promotes favorable vertical phase separation.
- This cooperative interface engineering boosts charge transport and suppresses recombination, enabling organic solar cells to achieve a 20.1% power conversion efficiency and 17.0% mini-modules.

**Supplementary Information:**

The online version contains supplementary material available at 10.1007/s40820-026-02325-2.

## Graphical abstract

**Figure:**
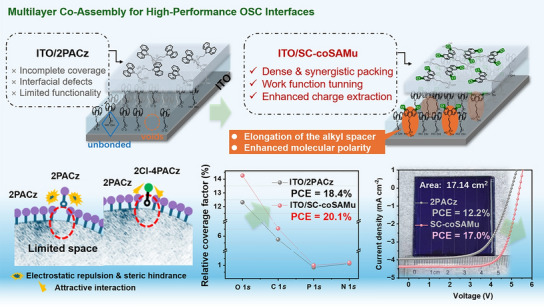

## Introduction

Self-assembled monolayers (SAMs) have emerged as powerful interfacial modifiers in organic and hybrid optoelectronic devices owing to their ability to tune energy-level alignment, enable selective charge extraction, and passivate interfacial defects [1, 2]. Among them, carbazole-based SAMs featuring phosphonic acid anchoring groups, such as 2PACz [(4-(7H-dibenzo[c,g]carbazol-7-yl)ethyl)phosphonic acid] [3], have been widely employed as hole-selective layers on indium tin oxide (ITO) electrodes [4]. These interlayers have contributed to power conversion efficiencies (PCEs) exceeding 20% [5–9] and enhanced operational stability in both perovskite and organic solar cells (OSCs) [10–14], thereby accelerating the development of photovoltaic technologies that are lightweight, mechanically flexible, semi-transparent, and compatible with scalable roll-to-roll manufacturing.

**Fig. 1:**
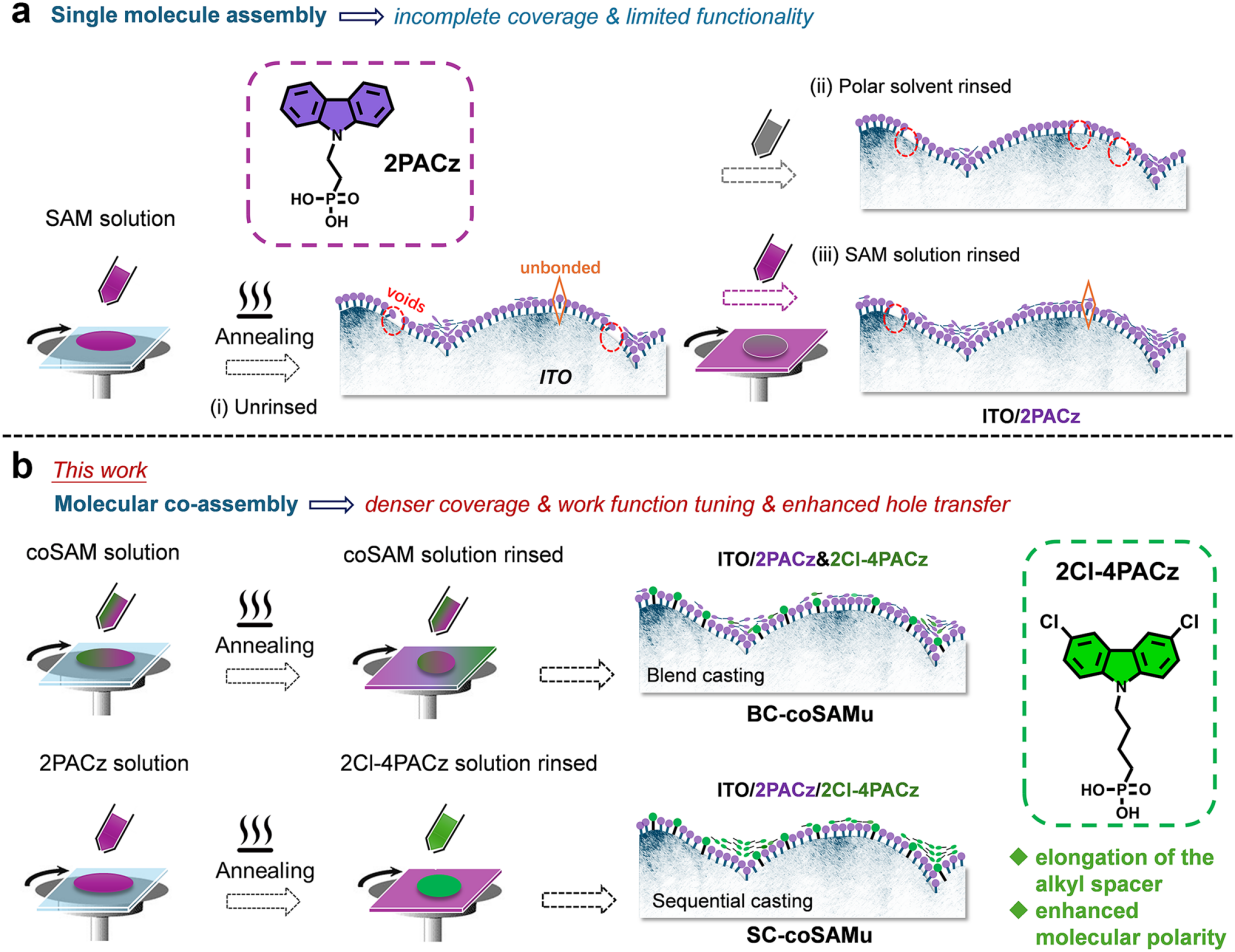
Schematic illustration of interface engineering evolution on the ITO surface:** a** conventional processing of a single SAM molecule to form a quasi-monolayer, and **b** molecular co-assembly of 2PACz and 2Cl-4PACz via blend casting and sequential casting

From the perspective of formation mechanisms, SAMs typically form uniform, monomolecular-thick functional layers via chemisorption. Their phosphonic acid head groups covalently bond with oxide surfaces, ensuring stable attachment at a molecular level [1, 15]. With this characteristic in mind, numerous studies have focused on optimizing monolayer structures. It is widely accepted that, in principle, quasi-monolayer or few-molecule-thick multilayers can achieve complete surface coverage and provide optimal interfacial functionality [16–19]. However, under typical solution-processing conditions, producing defect-free SAMs remains challenging due to both process- and material-related factors. Substrate roughness, incomplete molecular adsorption, and variations in post-deposition rinsing can result in incomplete ITO coverage (Fig. 1a), leading to fluctuations in device performance [20–23]. Meanwhile, homogeneous SAMs composed of a single type of molecule are limited by intrinsic molecular properties: small molecules are prone to uneven deposition, aggregation, and poor interfacial compatibility [24, 25]. These imperfections can degrade hole-transport layer (HTL) quality, increase interfacial recombination, impede charge extraction, and ultimately reduce OSC efficiency [23–26].

Moreover, recent studies have revealed that SAM assembly is governed, not only by anchoring interactions with the substrate, but also by intermolecular interactions within the monolayer [1, 4]. Upon adsorption, the system undergoes a transition from a disordered three-dimensional molecular mixture to a quasi-two-dimensional ordered packing. This process can be precisely tuned by engineering the strength and specificity of intermolecular interactions [1]. On this basis, the strategy of co-assembled SAMs (coSAMs) has emerged: by combining two complementary molecules, the interfacial composition and morphology can be synergistically optimized, enabling simultaneous improvements in device performance and functional versatility [24, 27–30]. Several studies have validated this strategy. For instance, Sun et al. demonstrated that introducing 1-hydroxybenzotriazole (HOBT) into (4-(7H-dibenzo[c,g]carbazol-7-yl)butyl)phosphonic acid (4PADCB) significantly improved molecular packing and film uniformity, leading to a PCE of 19.66% [31]. Similarly, Li et al. reported that incorporating the small molecule PyCA-3F at the buried interface between 2PACz and perovskite/organic layers effectively suppressed 2PACz aggregation, while simultaneously improving surface smoothness and energy-level alignment [21]. Moreover, rational design of bifunctional anchoring groups (e.g., carboxylic acid and phosphonic acid combinations) has been shown to strengthen molecule–substrate binding, while tailored energy-level arrangements and intermolecular π-π stacking further promote carrier transport and thermal stability [32].

Compared with single-component SAMs, coSAMs provide greater flexibility in tuning molecular packing density and interfacial electrostatic potential, thereby enabling more effective interfacial passivation and controllable charge dynamics. Here we propose a coSAMs strategy that employs structurally similar molecules with different alkyl chain lengths to achieve complementary filling, thereby eliminating interfacial voids and forming a dense, multifunctional interfacial layer. Specifically, we exploit the cooperative assembly of 2PACz and its chlorine-substituted analogue (4-(3,6-dichloro-9H-carbazol-9-yl)butyl)phosphonic acid (2Cl-4PACz) [20], both featuring a carbazole core connected via an alkyl linker (ethyl in 2PACz and butyl in 2Cl-4PACz) to a phosphonic acid anchoring group (Fig. 1b). These two components exhibit synergistic interactions: in 2Cl-4PACz, chlorine atoms substituted at the 3- and 6-positions of carbazole introduce a surface-oriented dipole that shifts the ITO work function (WF) toward more favorable hole injection, thereby improving hole extraction. Meanwhile, their vertical coordination combines favorable π-π stacking with reduced steric hindrance, which enhances electronic coupling at the electrode interface and achieves tighter molecular packing with increased ITO surface coverage. In addition, the observed hole density localized on the SAM molecules (at the SAM/PM6 interface) suggests that they act as active intermediate states facilitating hole transfer upon light absorption. In this way, coSAMs not only tune electrode WFs but also contribute directly to interfacial charge-transfer (CT) processes through their excited-state electronic structures.

It is found that these carbazole-based molecules can form either conventional monolayers or well-organized self-assembled multilayers (SAMu) [17] depending on the deposition conditions (Fig. 1a). In the sequential-casting (SC) approach, 2PACz is first deposited on ITO to form a bottom monolayer, followed by 2Cl-4PACz as an upper layer, with a minor fraction penetrating the underlying layer to chemisorb onto ITO surface. The 2PACz-rich bottom monolayer primarily governs energy-level alignment and WF modulation, which is further enhanced by the surface dipole of 2Cl-4PACz. Meanwhile, the post-deposited upper-layer (2Cl-4PACz) complementarily inserts into interfacial voids and improves defect passivation. The incorporation of 2Cl-4PACz also promotes molecular packing and induces a more favorable vertical composition profile of the active layer near the HTL, thereby strengthening electronic coupling with the donor. These synergistic structural and electronic effects enable efficient charge transport and collection while suppressing trap-assisted recombination. As a result, representative PM6:L8-BO and D18:L8-BO devices incorporating SC-coSAMu exhibit PCEs of up to 20.1% with fill factors (FFs) exceeding 81%, outperforming devices with pristine 2PACz. Beyond small-area devices (0.042 cm^2^), a 17.14 cm^2^ SC-coSAMu-based mini-module delivers a PCE of 17.0%, representing a 39% increase over its 2PACz-based counterpart (12.2%), demonstrating the scalability of the coSAMs strategy for large-area devices. This work redefines the structural paradigm of SAM interlayers in OSCs, demonstrating that controlled multilayered co-assembly offers a versatile and scalable route toward high-efficiency and stable optoelectronic devices through molecular-level interfacial engineering.

## Experimental Section

### Materials

2Cl-4PACz was synthesized according to the previous report [20]. 2PACz was purchased from Henan Alfa Chemical Co., Ltd. PM6, L8-BO, and PDINN were purchased from Solarmer Materials Inc. All chemicals and solvents purchased from Sigma-Aldrich were of reagent grade and used directly.

### Device Fabrication

OSC devices were fabricated on ITO glass substrates with the conventional structure of ITO/HTL/Active layer/PDINN/Ag. The patterned ITO-coated glasses were cleaned with detergent and then underwent a wet-cleaning process in an ultrasonic bath, followed by rinsing with ultrapure water, acetone, and isopropanol in sequence and then dried in an oven overnight to remove the residual solvents. Such pre-cleaned ITO substrates were treated with UV-ozone for 25 min, on which then diluted ethanol solution of various SAMs with a concentration of 0.3 mg mL^−1^ was spin-coated at 3000 rpm for 30 s, and thermally annealed at 65 °C for 5 min under air atmosphere. Subsequently, the SAM solutions were spin-cast twice at 6000 rpm for 30 s each. Afterward, they were transferred into a nitrogen-purged glove box for further use. The optimized binary PM6:L8-BO (1:1.2, weight ratio) blend was dissolved in chloroform (CF) with a total solid content of 15.4 mg mL^−1^ for 1 h at 45 °C. Before the spin-coating process, 0.75% 1-chloronaphthalene (v/v) was added to the CF solutions. The blend solution was spin-cast at 3000 rpm for 30 s over HTL layer on the ITO glasses to form an active layer with a thickness of around 100 nm. Subsequently, the blend films were heated at 85 °C for 5 min. Then, the methanol solution of PDINN with a concentration of 1 mg mL^−1^ was spin-coated over the active layers at 3000 rpm for 30 s. Finally, a 100 nm Ag layer was deposited on top of the PDINN under vacuum at a pressure of ca. 4 × 10^–4^ Pa through a shadow mask for controlled solar illuminating area of ~ 0.042 cm^2^.

### Module Fabrication

Solar mini-modules were fabricated with the device structure ITO/HTL/PM6:L8-BO/PNDIT-F3N/Ag on patterned substrates (50 mm × 50 mm). As shown in Fig. S18, a picosecond green laser (532 nm) was used to define the P1, P2, and P3 scribing lines (0.2 mm wide). The PM6:L8-BO active layer and PNDIT-F3N electron transport layer were deposited under the same conditions as those used for the small-area devices, followed by thermal evaporation of the Ag electrode. The final module consisted of six series-connected subcells with a total area of 17.64 cm^2^. The interconnection dead zone width was approximately 0.2 mm, resulting in an active area of 17.14 cm^2^ and a geometric fill factor of 97.1%.

## Results and Discussion

### Cooperative Molecular Interactions in coSAMs

**Fig. 2:**
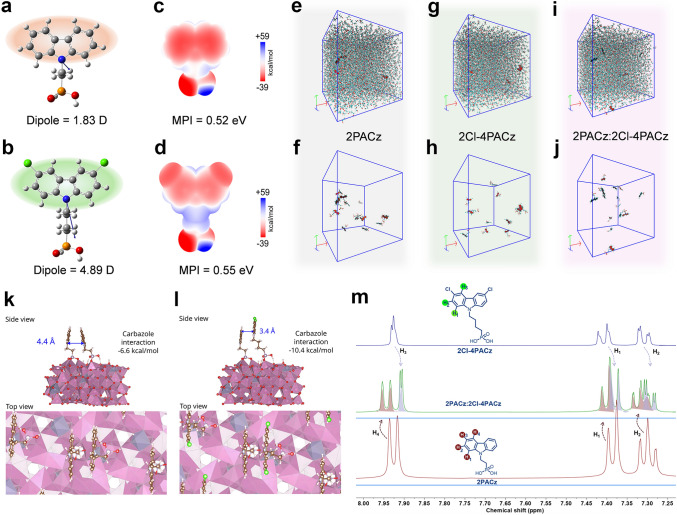
**a, b** Molecular structure and dipole moment vector, **c, d** EPS and the respective molecular polarity index (MPI) of 2PACz and 2Cl-4PACz. Final snapshots (t = 100 ns) of MD simulations of SAMs in ethanol for **e** 2PACz, **g** 2Cl-4PACz, and **i** a 1:1 mixture of 2PACz and 2Cl-4PACz. Panels **f**, **h**, and **j** show the corresponding frames with ethanol molecules omitted for clarity. All systems exhibit the formation of small aggregates, with the 1:1 mixture displaying preferential aggregation between chlorinated and non-chlorinated molecules. Optimized adsorption geometries of **k** 2PACz_2PACz and **l** 2PACz_2Cl-4PACz on ITO (111) surface, illustrating inter-carbazole distances and calculated interaction energies between adsorbers. **m** ^1^H NMR spectra of 2PACz, 2Cl-4PACz, and their mixture in the C_2_D_5_OD

SAMs were formed on hydrophilic ITO surfaces via spin-coating, using three commonly reported methods (Fig. 1a): (i) spin-coating followed by annealing [7, 21, 33]; (ii) after process (i), rinsing twice with a polar solvent (e.g., methanol or ethanol) [5, 14, 20]; (iii) after process (i), rinsing twice with the SAM solution [11, 12, 34]. Method (iii) was adopted in this work. A reference SAMu of 2PACz was prepared by spin-coating onto UV-ozone-treated ITO, followed by mild annealing at 65 °C for 5 min and rinsing with a 2PACz stock solution. For coSAMus, two strategies were employed: blend casting (BC) and sequential casting (SC) (Fig. 1b). In the BC method (for BC-coSAMu), a mixed solution of 2PACz and 2Cl-4PACz (at the optimal weight ratio of 10:3, determined from device performance; Fig. S1, Table S1) was deposited using the same procedure as method (iii). In the SC method (for SC-coSAMu), a 2PACz layer was first deposited, then rinsed with a 2Cl-4PACz solution. The intrinsic electronic properties of 2Cl-4PACz and its role in interface engineering were investigated using density functional theory (DFT), as described in Supporting Information. Substituting two hydrogen atoms on the carbazole moiety with chlorine atoms increased both the magnitude and spatial separation of atomic charges (Fig. 2a, b). In addition, elongation of the alkyl spacer increased the distance between an electron-rich carbazole core and an electron-deficient phosphonic acid group, as revealed by Mulliken population analysis (Table S2). Together, these structure modifications contribute to a higher dipole moment for 2Cl-4PACz (4.89 D) compared with 2PACz (1.83 D). Moreover, electrostatic potential surface (EPS) analysis [35] revealed a greater molecular polarity index (MPI) for 2Cl-4PACz (0.55 eV) than that for 2PACz (0.52 eV) (Fig. 2c, d), suggesting increased molecular polarity. The introduction of electron-withdrawing chlorine atoms also stabilized the highest occupied molecular orbital (HOMO) level by shifting electron density toward the molecular periphery, thereby reducing intramolecular repulsion (Fig. S2).

Solid-state DFT simulations were performed for 2PACz and 2Cl-4PACz molecules adsorbed on ITO (111) surface to probe their intermolecular interactions (Fig. 2k, l) and to investigate their effects on the WF of ITO [3]. The calculated intermolecular interaction energies between carbazole units revealed that the 2PACz_2Cl-4PACz system exhibited stronger π-π stacking interactions (− 10.4 kcal mol^−1^) than the 2PACz_2PACz system (− 6.6 kcal mol^−1^), which can be attributed to a shorter stacking distance, i.e., 3.4 versus 4.4 Å. This reduced intermolecular distance enhances non-covalent interactions, providing a potential driving force for denser SAM packing on ITO surfaces. An interesting aspect of these interactions is that they resemble those observed prior to deposition, while still in the ethanol solution. We investigated the behavior of the SAMs in solution using molecular dynamics (MD) simulations, modeling both the pure SAMs in ethanol and their 1:1 mixture. Simulation details are provided in the Supporting Information. Aggregate formation was observed in all simulations; however, in the mixed system, persistent heteromolecular aggregation was particularly pronounced. This is evidenced by a significantly higher number of close contacts (< 4 Å) between 2PACz and 2Cl-4PACz compared to other homomolecular contacts (Figs. 2e–j and S3). Representative dimer structures from the mixed simulation reveal π-π stacking between carbazole units, O–H interactions involving anchoring groups, and combined motifs in which both interactions occur simultaneously (Fig. S4), highlighting complementary interaction pathways absent in the single-component systems. These findings demonstrate that cooperative molecular organization begins in solution, providing a mechanistic basis for the denser, more cohesive SAMu layers observed on ITO.

To experimentally validate these molecular interactions, ^1^H NMR spectroscopy (Fig. 2m) was performed on a 1:1 (w/w) mixture of 2PACz and 2Cl-4PACz in C_2_D_5_OD. Relative to the spectra of the individual compounds, the mixed system exhibited pronounced chemical shift variations: the aromatic resonances of 2Cl-4PACz shifted upfield, whereas those of 2PACz shifted downfield, indicating specific intermolecular interactions within the blend. In neat 2PACz solution, the aromatic protons appeared at relatively high-field positions, reflecting weak 2PACz_2PACz interactions. Upon mixing with 2Cl-4PACz, the downfield shift of 2PACz aromatic protons denotes reduced shielding, most plausibly due to an electron-density transfer from 2PACz to 2Cl-4PACz through π-π interactions. The chlorine substituents may further enhance this deshielding through inductive effects. Collectively, these observations demonstrate that the intrinsic 2PACz_2PACz interactions are strengthened in the presence of 2Cl-4PACz, and that well-defined π-π stacking motifs are established between the two molecules. Such reinforced interactions are well aligned with the DFT simulation results and are expected to promote denser molecular packing in the blended 2PACz:2Cl-4PACz films, thereby enhancing structural order and improving the film quality of the SAM-based hole-extraction layer on ITO.

### Formation of Dense and Ordered Multilayer SAM Architectures

**Fig. 3:**
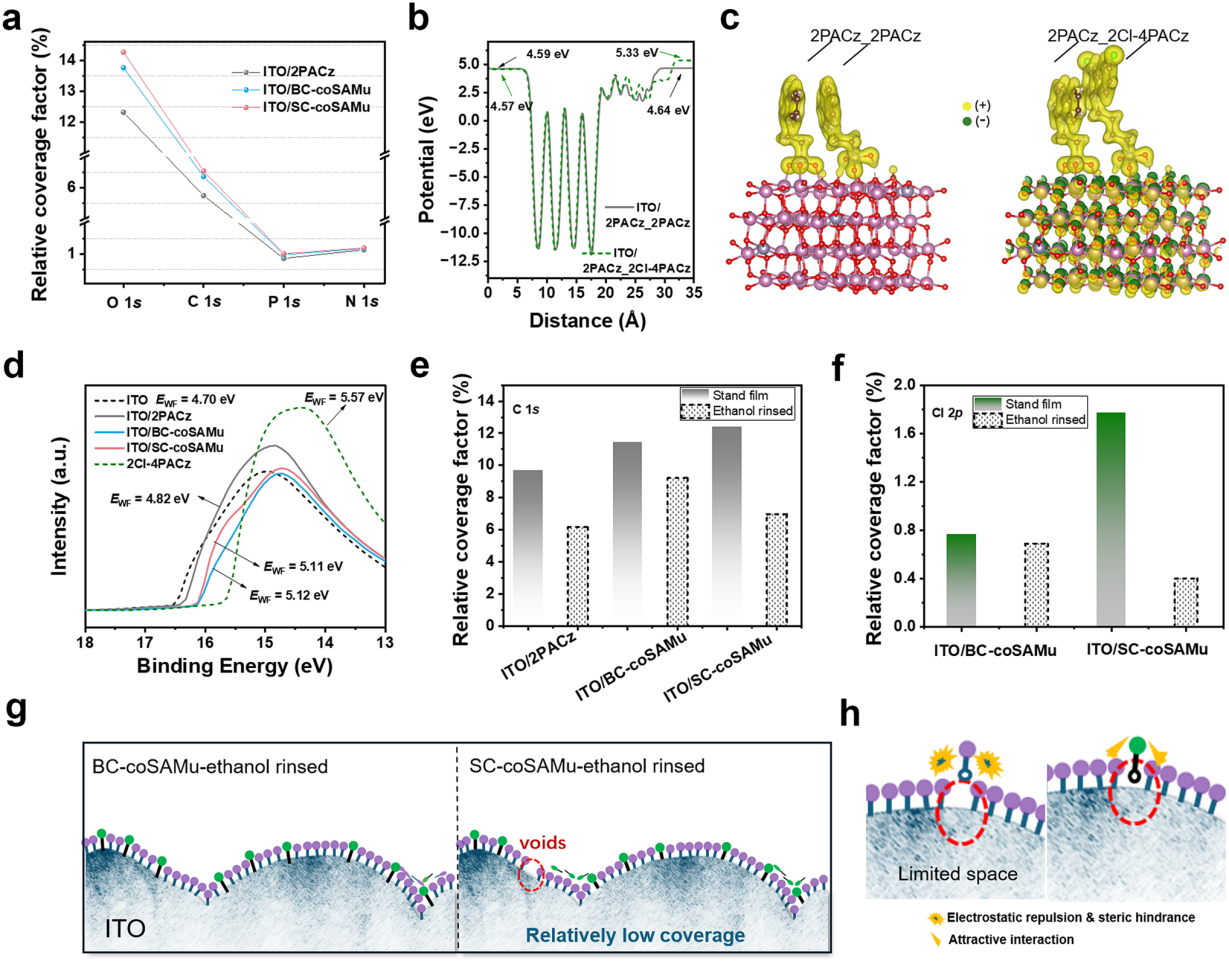
**a** RCF for SAMu-modified ITO surfaces, calculated by normalizing the core-level areas of C 1*s*, O 1*s*, N 1*s*, and P 2*p* to that of In 3*d*_3/2_. **b** Calculated work functions for the two SAM/ITO systems, with Fermi level shifted to zero. **c** Charge difference between 2PACz_2PACz/ITO and ITO (left) and between 2PACz_2Cl-4PACz/ITO and ITO (right), with positive and negative densities represented by yellow and green colors, respectively. **d** UPS spectra (He I lamp with a photon energy of 21.22 eV) of bare and SAMs-modified ITO surfaces for direct determination of WF. **e, f** RCF for SAMu-modified ITO surfaces, calculated by normalizing the core-level areas of C 1*s* and Cl 2*p* to the In 3*d*_3/2_ core-level area. **g** Schematic illustration of SAM distributions for BC-coSAMu and SC-coSAMu on ITO glass after ethanol rinsing. **h** Schematic illustration of defect filling by short-chain 2PACz and preferential chemisorption of long-chain 2Cl-4PACz on ITO

To further evaluate the quality and coverage of SAMu on ITO surface, X-ray photoelectron spectroscopy (XPS) was conducted [3, 33]. Core-level spectra of C 1*s*, O 1*s*, N 1*s*, and P 2*p* were collected and normalized to the In 3*d*_3/2_ signal from the substrate (Figs. S5 and S6, Table S3). The relative coverage factor (RCF) [3, 5] indicated a clear trend in surface coverage: 2PACz < BC-coSAMu < SC-coSAMu (Fig. 3a, Table S3), indicating progressively improved molecular coverage as a result of co-assembly. Beyond the quantitative increase in coverage, further insights were obtained by analyzing the chemical environment of each element. Notably, from 2PACz to BC-coSAMu and SC-coSAMu, the XPS spectra of all detected elements exhibited systematic shifts toward higher binding energy (Figs. S6 and S7). This shift, particularly evident in Cl-related signals, indicates reduced electron density around the Cl atoms, consistent with stronger intermolecular interactions and increased electronic polarization within the SAM. These spectral changes support the hypothesis that introducing a highly polar Cl-substituted analogue (2Cl-4PACz) strengthens intermolecular coupling, plausibly through dipole–dipole and π-π interactions among carbazole groups. Taken together, these results confirm that the enhanced surface coverage observed in coSAMus arises, not merely from increased material deposition, but from improved molecular arrangement driven by stronger intermolecular forces. This denser and more uniform molecular packing (as confirmed by atomic force microscopy (AFM) measurements, discussed below) is in strong agreement with the results from earlier DFT simulations and ^1^H NMR studies. Such structural improvements are expected to yield superior interfacial passivation and facilitate stronger electronic coupling at the electrode interface. Ultimately, the formation of a compact and electronically coherent SAM or SAMu contributes to more efficient hole extraction from the bulk heterojunction (BHJ) active layer, which is critical for high-performance organic photovoltaic devices.

### Interfacial Dipole Engineering and Work Function Tuning

Furthermore, as shown in Fig. 3b, the DFT-calculated WF of the ITO/2PACz_2PACz system was 4.64 eV, while that of the ITO/2PACz_2Cl-4PACz configuration increased to 5.33 eV. This theoretical trend agrees with ultraviolet photoelectron spectroscopy (UPS) measurements: the WF of modified ITO substrates increased from 4.82 eV for pristine 2PACz to 5.12 eV for BC-coSAMu and 5.11 eV for SC-coSAMu (Fig. 3d). The enhanced WF in coSAMu is attributed to the stronger surface dipole introduced by the electron-withdrawing 2Cl-4PACz, which induces a larger vacuum-level shift via interfacial polarization. This leads to improved energetic alignment with the PM6 HOMO and a reduced hole-extraction barrier at the anode interface (Fig. S8). Figure 3c shows electron-density redistribution upon SAM adsorption by comparing each ITO/SAM system to a bare ITO reference. In the charge-difference plots, yellow regions represent areas of electron depletion (positive charge), while green regions denote electron accumulation (negative charge). These distributions demonstrate significant interfacial electronic rearrangement driven by the adsorbed molecules. In particular, the ITO/2PACz_2Cl-4PACz system shows a more pronounced effect than the ITO/2PACz_2PACz system, which is attributed to the larger intrinsic dipole moment of 2Cl-4PACz. This stronger dipole polarizes the interface more effectively, drawing electron density toward the molecule (green lobes) and leaving regions of the ITO surface relatively more electron-deficient (yellow lobes). This enhanced interfacial polarization results in a greater WF shift for the 2Cl-4PACz-containing system, leading to more efficient hole extraction from the active layer to anode. Such improved alignment minimizes energetic offsets and potentially contribute to higher FFs and open-circuit voltages (*V*_oc_).

### Defect Filling at Buried Interfaces

However, an intriguing discrepancy arises from the analysis of Cl 2*p* XPS signals (Fig. S7 and Table S3). At a 30% doping ratio, the SC method achieved nearly twice the RCF of the BC method (Fig. 3f), suggesting that the SC method enables more effective incorporation of 2Cl-4PACz into the ITO surface. Despite this higher surface loading, the WFs of the two coSAMu-modified ITO samples remain comparable (Fig. 3d). To probe this inconsistency, we examined the inner structure of the monolayers. Both coSAMu films (via BC and SC methods) were subjected to ethanol rinsing (6000 rpm, twice) to remove physisorbed molecules, retaining only strongly bound SAM species typically oriented perpendicularly to the substrate [1, 5]. XPS characterization of the washed films (Figs. S9–S11, Table S4) provided insights into the effective monolayer composition and orientation. Compared to the untreated multilayer films, the ethanol-rinsed washed samples exhibited a general decrease in molecular coverage, as evidenced by reduced C 1*s* RCF values (Fig. 3e). Notably, a greater fraction of SAMs was removed from the SC-derived films, compared with the BC-derived ones. In particular, the RCF of Cl 2*p* decreased by up to 77%, suggesting that a substantial fraction of 2Cl-4PACz molecules, presumably those not chemically bonded to the ITO surface, was washed away (Fig. 3f). These molecules are likely located in the upper region of the 2PACz monolayer. However, approximately 23% of the Cl 2*p* signal remained, implying that some 2Cl-4PACz molecules had penetrated defect sites within the 2PACz layer, potentially passivating these defects.

Notably, the SC-derived monolayer exhibited lower surface coverage than that obtained via the BC method, indicating that a pure 2PACz SAM achieves lower surface coverage than a SAM formed from 2Cl-4PACz:2PACz blended solution (Fig. 3g). This supports the hypothesis that under the SC approach, 2Cl-4PACz molecule can infiltrate defect sites within the 2PACz layer. However, only a limited fraction of 2Cl-4PACz forms stable chemical bonds with ITO, while the majority remains weakly physisorbed. Because the intrinsic number of interfacial defects is limited, most 2Cl-4PACz molecules cannot chemically anchor. Among the three ethanol-processed monolayers, the 2PACz-only film exhibited the lowest C 1*s* RCF value. As surface molecular bonding nears saturation, repulsive interactions dominate, causing shorter 2PACz molecules to be displaced during rinsing, while longer 2Cl-4PACz molecules preferentially occupy the exposed sites via favorable π–π stacking and reduced steric hindrance (Fig. 3h), thus enhancing SAM packing density and film quality. Interestingly, the relative Cl 2*p* to C 1*s* RCF ratio in the monolayer films was similar for both deposition methods, 7.5% for BC and 5.8% for SC, indicating comparable chlorine content at the surface. This correlation is consistent with the nearly identical WF values measured for their corresponding multilayer films (5.12 vs. 5.11 eV). In contrast, a twofold difference in Cl 2*p* intensity was observed in the multilayer films for SC- and BC-processed samples, despite their similar WFs. This suggests that only the Cl-containing 2Cl-4PACz molecules within the buried monolayer significantly influence the WF, while those in the outer layers contribute minimally. To further validate this hypothesis, a series of 2PACz films underwent three different post-treatments: untreated, rinsed with pure 2PACz solution, and washed with ethanol. UPS results revealed that the WF remained around 4.82 eV for both untreated and 2PACz- rinsed films, but slightly decreased to 4.78 eV after ethanol rinsing (Fig. S12). These findings confirm that WF modulation is predominantly governed by the strongly bound inner monolayer, with negligible contribution from loosely adsorbed upper-layer molecules.

### Photovoltaic Performance of OSCs and Mini-Modules

**Fig. 4:**
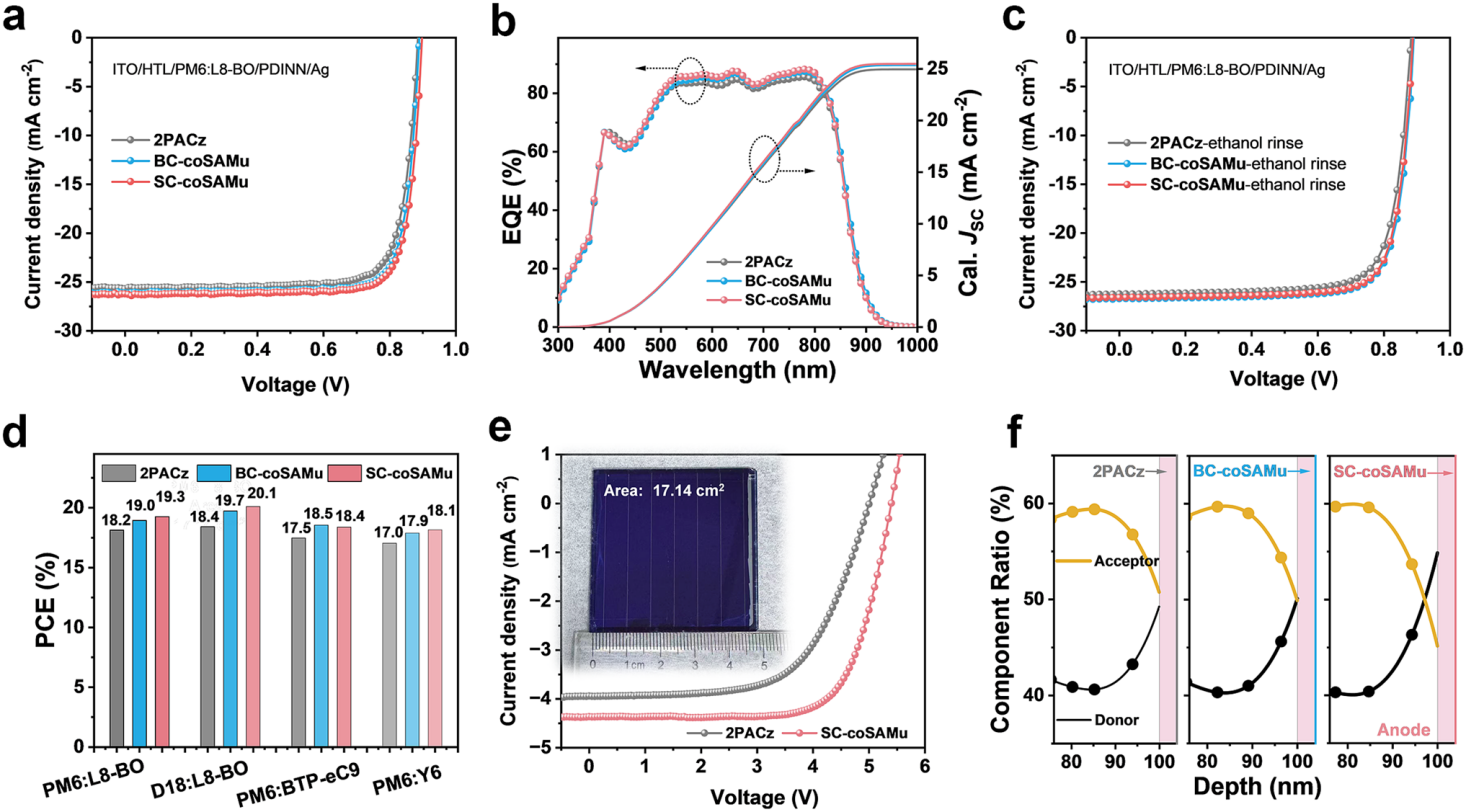
**a, c** *J-V* characteristics of PM6:L8-BO solar cells with different HTLs, measured **a** before and **c** after ethanol rinsing of 2PACz-, BC-, and SC-coSAMu layers. **b** EQE spectra of PM6:L8-BO solar cells with different HTLs, including 2PACz, BC-coSAMu, and SC-coSAMu. **d** Summary of photovoltaic parameters for OSCs based on different active layer systems, including PM6:L8-BO, PM6:BTP-eC9, D18:L8-BO, and PM6:Y6. **e** *J-V* characteristics of PM6:L8-BO mini-modules composed of six serially connected subcells, incorporating different HTLs (2PACz and SC-coSAMu). **f** Depth-resolved compositional profiles of PM6:L8-BO blend films deposited on 2PACz, BC-, and SC-coSAMu, obtained from depth-dependent absorption spectroscopy

To rationalize the role of co-assembled multilayers in modulating interfacial properties, we further investigated their formation and functionality through a comparative device study. Although 2Cl-4PACz alone exhibited suboptimal performance in previous studies, achieving efficiencies of only 14%, primarily due to its extended flexible alkyl spacer, which increases H···H interactions and thereby raises both the tunneling distance and the interfacial tunneling barrier [20], its incorporation into coSAMus with 2PACz leads to a significant enhancement in device performance. OSCs with a conventional device configuration of ITO/HTL/PM6:L8-BO/PDINN/Ag were fabricated. The current–voltage (*J-V*) characteristics of the optimized devices, along with the corresponding photovoltaic parameters, are summarized in Fig. 4a and Table 1. Using the BC method, with the ratios of 2Cl-4PACz:2PACz varying from 0:1 to 0.6:1 (w/w), the PM6:L8-BO-based devices achieved the best performance at the ratio of 0.3:1, with a PCE of 18.9%, a *V*_oc_ of 0.891 V, a short-circuit current density (*J*_sc_) of 26.14 mA cm^−2^, and a FF of 81.3% (Fig. S1 and Table S1). Applying the SC method further improved its PCE to 19.3% with a *V*_oc_ of 0.899 V, *J*_sc_ of 26.29 mA cm^−2^, and FF of 81.5%. Notably, both coSAMu-based devices obtained FFs exceeding 81%. In contrast, the 2PACz-control device delivered a moderate PCE of 18.1%, primarily due to slightly reduced *J*_sc_ (25.73 mA cm^−2^) and FF (79.7%) values. Device performance was further verified by fabricating 15 independent OSC devices for each condition (Fig. S13). The coSAMu-based devices also exhibited improved long-term stability compared to the 2PACz-based device, as shown in Fig. S14 under maximum power point tracking for 240 h. In addition, the *J*_sc_ values calculated from external quantum efficiency (EQE) measurements aligned well with those from *J-V* measurements, with deviations within 4% (Fig. 4b), confirming the reliability of the results.

**Table 1:** Photovoltaic performance of the OSCs based on PM6:L8-BO under the illumination of AM 1.5 G, 100 mW cm^−2^

HTL	*V*_oc_ [V]	*J*_sc_ [mA cm^−2^]	Cal. *J*_sc_^d^ [mA cm^−2^]	FF [%]	PCE_max_ [%]
2PACz^a^	0.887	25.73	24.95	79.7	18.2 (17.9 ± 0.18)^e^
BC-coSAMu^a^	0.892	26.14	25.36	81.3	19.0 (18.7 ± 0.13)^e^
SC-coSAMu^a^	0.899	26.29	25.48	81.5	19.3 (19.1 ± 0.15)^e^
SC-coSAMu^b^	0.910	27.13	26.29	81.4	20.1 (19.8 ± 0.29)^e^
2PACz^c^	5.003	3.94	−	61.9	12.2 (11.9 ± 0.33)^f^
SC-coSAMu^c^	5.399	4.37	−	72.0	17.0 (16.7 ± 0.27)^f^

^a^Device area:0.042 cm^2^. ^b^Device based on D18:L8-BO ^c^Device area:17.14 cm^2^. ^d^Integral *J*_sc_ calculated from the EQE curves. ^e^The average PCE values in brackets were obtained from 15 devices. ^f^The average PCE values in brackets were obtained from 5 devices

The photovoltaic performance of monolayer-based hole-transport layers (HTLs), obtained via ethanol rinsing, was also assessed (Fig. 4c and Table S5). Since thermal annealing of the active layer can affect the underlying HTL, the substrates were first annealed at 100 °C for 5 min prior to ethanol rinsing to obtain the well-defined monolayers of the respective HTLs. Compared with their multilayer counterparts, the rinsed monolayers exhibited slightly higher *J*_sc_ but lower FF, resulting in a marginal drop in overall efficiency. Among them, the BC-coSAM monolayer yielded the best performance, consistent with its higher surface coverage and improved interfacial passivation. Reversing the deposition order in the SC method, by depositing of 2Cl-4PACz before 2PACz, led to a severe drop in efficiency (PCE of only 14.3%, Fig. S15, Table S6). Similarly, BC-coSAMs with high 2Cl-4PACz content (at the ratio of 0.6:1) also performed poorly. These results underscore the crucial importance of vertical molecular arrangement in SAM design. Owing to its longer alkyl spacer, 2Cl-4PACz should be positioned above the more compact 2PACz layer to minimize tunneling barriers and facilitate efficient hole extraction [20]. This ordering ensures optimal energetic alignment and highlights the role of dipole orientation and molecular order in determining device performance. Importantly, the coSAM strategy was further validated across multiple active-layer systems, including PM6:BTP-eC9, D18:L8-BO, and PM6:Y6 (Fig. S16, Tables S7–S9). In all cases, coSAMu incorporation enhanced device performance (Fig. 4d), primarily through enhanced FF, demonstrating the broad applicability of the co-assembly approach. Notably, for the D18:L8-BO system, BC- and SC-coSAMu devices achieved champion PCEs of 19.7% and 20.1%, respectively (Fig. S16b, Table S7), corresponding to a 7%–9% improvement over 2PACz-only devices. These results highlight not only the efficiency gains but also the practical simplicity of incorporating 2Cl-4PACz as a universal strategy for optimizing HTLs in high-performance OSCs. Additional chlorine-substituted SAMs with different spacer lengths were incorporated into the 2PACz-based HTL using both BC and SC methods (Fig. S17, Tables S10–S12). As shown in Table S12, the C3 analogue (2Cl-3PACz) led to reduced device performance. This is mainly because the propyl spacer orients the carbazole head group roughly parallel to the plane of the PO_3_H_2_ anchoring group [5], introducing steric hindrance that weakens intermolecular interactions with 2PACz and limits void filling. In contrast, the C5 analogue (2Cl-5PACz) still improved performance relative to pristine 2PACz, although less effectively than 2Cl-4PACz, likely due to stronger intermolecular interactions between 2Cl-4PACz and 2PACz, arising from the more vertical orientation of the carbazole group [5]. These results further support the effectiveness of the complementary SAM strategy.

To further evaluate the scalability of these HTLs for large-area organic photovoltaic devices, a module consisting of six serially connected subcells was fabricated by spin-coating the chloroform solution of PM6:L8-BO onto ITO/2PACz and ITO/SC-coSAMu substrates (Figs. 4e and S18; Table 1). For a total active area of 17.14 cm^2^, the SC-coSAMu-based module delivered an outstanding PCE of 17.0%, whereas the 2PACz-based counterpart reached only 12.2%. This discrepancy primarily arises from the degradation in all three key parameters, particularly FF. As shown in Tables 1 and S7–S9, in small-area devices (0.042 cm^2^), the FF difference among these SAM-based HTLs is negligible (~ 2%). However, scaling up to 17.14 cm^2^ reveals a significant 12% gap in FF emerges between 2PACz and SC-coSAMu (Table 1). Such a discrepancy originates from increased defect accumulation in large-area devices: non-uniform interfaces—particularly at HTL/active-layer junctions—promote trap formation and recombination, ultimately leading to a pronounced decline in FF and overall device performance.

### Interfacial Morphology and Vertical Phase Optimization

Furthermore, we investigated how incorporating 2Cl-4PACz into the SAMu structure affects the surface morphology of HTL. AFM analysis (Fig. S19) reveals that all three HTLs possess comparable surface roughness; however, the 2Cl-4PACz-rich HTLs exhibit progressively increased water contact angles from 2PACz to BC- and SC-coSAMu (Fig. S20). This enhanced hydrophobicity indicates improved coverage by the chlorine-substituted SAMs and suggests stronger interfacial compatibility with the organic photoactive layer [24, 36]. Given that interface characteristics can modulate BHJ organization, film-depth-dependent light absorption spectrometry (FLAS) was employed to study vertical phase separation in PM6:L8-BO films (Fig. S21). Compared to the pristine 2PACz case, devices utilizing coSAMu exhibited a more uniform vertical distribution of donor and acceptor components (Fig. S21d–f), minimizing compositional fluctuations. Moreover, SC-coSAMu induces slight PM6 enrichment near the HTL (depth: 80–100 nm) (Fig. 4f), which favors efficient hole injection and further mitigates interfacial recombination. Such vertical homogeneity and donor accumulation enhance exciton dissociation, facilitate balanced charge transport, and suppress recombination losses, factors contributing to the observed increase in FF from 78% to over 81%. To assess whether SAMu modification perturbs the BHJ microstructure, grazing-incidence wide-angle X-ray scattering (GIWAXS) measurements were conducted on the upper BHJ layers (Figs. S22–S24, Table S13) [37, 38]. All blends exhibited nearly identical in-plane lamellar (100) and out-of-plane π-π stacking (010) diffraction features, along with comparable coherence lengths, confirming that the SAMu does not significantly disrupt the molecular packing within the active layer. Overall, the morphology of the upper BHJ remains largely unchanged, indicating that PV performance improvements originate primarily from HTL surface and interface modification rather than from alterations in bulk BHJ ordering.

### Charge Extraction and Recombination Suppression Mechanisms

**Fig. 5:**
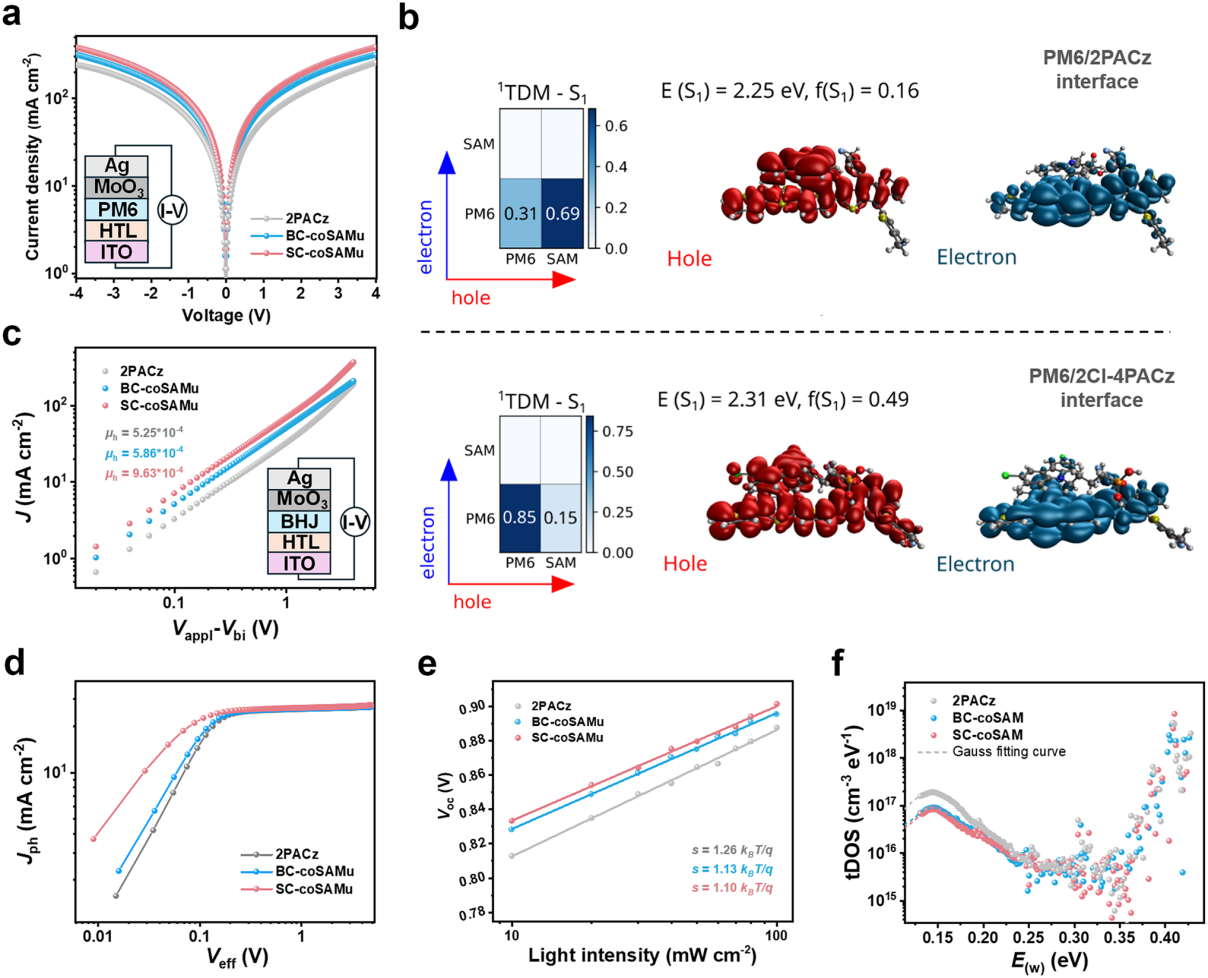
**a** Dark current characteristics of hole-only devices employing 2PACz, BC-, and SC-coSAMu as HTLs, respectively; inset: schematic of the hole-only device architecture. **b** Properties of first low-lying singlet excited state *S*_1_ for PM6/2PACz and PM6/2Cl-4PACz interfaces, visualized via ^1^TDM, distinguishing local and CT excitations. **c** *J-V* characteristics of hole-only PM6:L8-BO devices with different HTLs. **d** *J*_ph_ versus *V*_eff_, **e** light-intensity dependence of *V*_oc_, **f** trap density of states, for PM6:L8-BO devices incorporating BC- and SC-coSAMu HTLs, respectively

The effects of 2Cl-4PACz modification on device operation mechanisms, including charge transport, recombination, and extraction, were systematically evaluated. First, we investigated how functionalizing 2PACz with 2Cl-4PACz affects hole-injection from the ITO electrode into the organic semiconductor. For this purpose, hole-only devices based on the donor polymer PM6 were fabricated (Fig. 5a). Devices incorporating coSAMu-modified electrodes exhibited slightly higher current densities than that using pristine 2PACz, indicating improved Ohmic contact at the HTL/semiconductor interface. Subsequently, hole mobility (*μ*_h_) was quantified using the space-charge-limited current (SCLC) method [39] (Fig. 5c). The measured mobility values were higher for the SC-coSAMu-based devices (8.63 × 10^−4^ cm^2^ V^−1^ s^−1^) than those from 2PACz-only (5.25 × 10⁻^4^ cm^2^ V^−1^ s^−1^) and BC-coSAMu-based (5.86 × 10^−4^ cm^2^ V^−1^ s^−1^) ones, further supporting the notion that 2Cl-4PACz improves hole-injection efficiency.

To understand the origin of this enhancement, the electronic structures at the HTL/PM6 interfaces were examined. Six representative configurations between the SAM molecules and the PM6 polymer were studied (Figs. S25 and S26). Geometry optimizations revealed dominant π-π stacking interactions between the carbazole units of the SAM molecule and the PM6 backbone, suggesting favorable electronic coupling. The maximum energy difference between the most and least stable configurations was 0.26 eV for 2PACz and 0.75 eV for 2Cl-4PACz, indicating greater configurational sensitivity in the chlorinated SAM molecule. This larger configurational energy variation in 2Cl-4PACz arises from chlorine-induced modulation of the SAM's electronic structure, which enhances its interaction with the benzo[1,2-c:4,5-c']dithiophene-4,8-dione (BDD) moiety in PM6. Moreover, in the lowest energy conformations, the carbazole moiety aligns via π-stacking with the electron-deficient BDD segment of PM6, forming a more complementary interaction with the electron-rich 2Cl-carbazole present in 2Cl-4PACz. These findings collectively highlight the role of chlorination in tuning interfacial interactions.

Going beyond ground-state properties of the SAM/PM6 interfaces, we further investigated the three lowest-lying singlet excited states of the most energetically favorable configurations. As illustrated in Figs. S27 and 5b, one-electron transition density matrix (^1^TDM) analysis was employed to distinguish between local excitations and charge-transfer (CT) characteristics. In the ^1^TDM, off-diagonal elements reflect electron-density transfer between the SAM and PM6 (indicative of CT transitions), while diagonal elements represent local excitations confined within either the SAM or PM6. Notably, the lowest singlet excited states (S_1_–S_3_) of both the 2PACz/PM6 and 2Cl-4PACz/PM6 interfaces comprise both local PM6 to PM6 excitations and charge-transfer (SAM to PM6) contributions. For the 2PACz/PM6 interface, the CT content ranges from 15 to 81% across S_1_–S_3_, while for the 2Cl-4PACz/PM6 interface it varies 35−69%. These results indicate substantial interfacial orbital hybridization, enabling partial hole delocalization across the SAM/PM6 boundary. The observed hole density on the SAM molecules further confirms their role as active intermediate states facilitating hole transfer upon light absorption. This enhanced electronic coupling likely promotes more efficient hole transfer from PM6 into the SAM, thereby adding a complementary functional role beyond simply tuning the ITO WF. In particular, light absorption at the SAM/PM6 interface can directly generate intermolecular CT states, highlighting that the excited-state electronic structure of SAMs plays an active role in interfacial charge-transfer processes.

To assess the functional impact of enhanced hole extraction, exciton dissociation and charge collection efficiencies were analyzed through the relationship between photocurrent density (*J*_ph_) and effective photovoltage (*V*_eff_) [40]. Figure 5d demonstrates that all three OSCs exhibit similar exciton dissociation efficiencies (*P*_diss_ = 98.2% for 2PACz, 98.6% for BC-coSAMu, and 98.8% for SC-coSAMu). However, devices based on coSAMu exhibited higher charge collection efficiencies (*P*_coll_ = 90.3% for 2PACz, 92.0% for BC-coSAMu, and 92.2% for SC-coSAMu), suggesting more effective carrier extraction in the coSAMu-modified systems. Furthermore, transient photocurrent (TPC) measurements provided additional insight into charge extraction dynamics (Fig. S28) [41]. Devices fabricated with SC-coSAMu method exhibited the shortest carrier sweep-out time (0.182 μs), compared with 0.187 μs for BC-coSAMu and 0.222 μs for 2PACz, indicating faster and more efficient charge extraction. Furthermore, the reduction in photoluminescence intensity for coSAMu-based films compared to 2PACz indicates more efficient charge extraction at the interface (Fig. S29). These observations correlate well with the enhanced *J*_sc_ and FF observed in PM6:L8-BO-based OSCs incorporating SC-coSAMu, underscoring the benefits of the improved interfacial engineering strategy.

To gain deeper insights into charge recombination kinetics, the dependence of *J*_sc_ on light intensity (*P*_light_) was examined using the relationship of *J*_sc_ ∝ (*P*_light_)^α^ [42], which provides a measure of bimolecular recombination. As illustrated in Fig. S30, α values for all three devices approached 1, indicating negligible bimolecular recombination. Additionally, the relationship between *V*_oc_ and *P*_light_, expressed as *V*_oc_∝n* k*_*B*_*T/q* ln(*P*_light_) [43], was evaluated, where *n* is the ideality factor ranging between 1 and 2, *k*_B_ is Boltzmann's constant, *q* stands for the elementary charge, and *T* represents the absolute temperature. Figure 5e shows that the slopes for devices based on 2PACz, BC-, and SC-coSAMu were 1.26, 1.13, and 1.10 *k*_B_*T*/*q*, respectively. This confirms that devices employing coSAMu as the HTL exhibit reduced trap-assisted recombination. Capacitance–frequency (*C*-*ω*) measurements were performed to determine the trap density of states (*N*_T_(*E*_*ω*_)) in the OSCs [44]. As displayed in Fig. 5f, the trap density for devices using 2Cl-4PACz-modified 2PACz was lower (≈ 9.16 × 10^16^ cm^−3^ eV^−1^ for BC-coSAMu and 8.23 × 10^16^ cm^−3^ eV^−1^ for SC-coSAMu) than those for devices using pure 2PACz (≈ 1.91 × 10^17^ cm^−3^ eV^−1^), further confirming reduced recombination. The higher trap density in the 2PACz-based device accounts for its lower efficiency in large-area OPV modules. Transient photovoltage (TPV) and charge extraction (CE) measurements under varying light intensities provided further insights into recombination dynamics (Fig. S31). Carrier lifetime (*τ*) was derived from TPV, while carrier density (*n*) was inferred from CE. The bimolecular recombination rate constants (*k*_rec_) were calculated using the formula: *k*_rec_ = ((*λ* + 1)*nτ*)^−1^ [45], where *λ* denotes the recombination order. Figure S31b displays *k*_rec_ as a function of n, showing that 2Cl-4PACz-modified OSCs maintained lower *k*_rec_ levels as n increased, while the pure 2PACz device exhibited a significant increase in *k*_rec_. This trend aligns with reduced recombination and improved FF in the PM6:L8-BO device incorporating coSAMu.

## Conclusions

In summary, this work presents a rational co-assembly strategy for constructing high-performance HTLs by combining two carbazole-based phosphonic acid molecules, 2PACz and its chlorine-substituted analogue 2Cl-4PACz, with distinct spacer lengths and polarities. Their synergy facilitated by favorable π–π stacking and reduced steric hindrance, effectively passivates interfacial defects and reconfigures molecular interactions at the ITO surface, yielding enhanced surface coverage and the formation of a densely packed, electronically coupled multilayer that upshifts the ITO WF. A key element to this strategy is the distinct functional division between layers: WF modulation is primarily governed by the bottom chemisorbed monolayer (bound buried SAM region) on the metal oxide surface, while the upper-layer molecules (loosely adsorbed SAM region) exert minimal influence on overall BHJ morphology but play a critical role in strengthening interfacial compatibility with the donor polymer and promoting favorable vertical phase separation of the active layer near the HTL. Sequential casting of 2Cl-4PACz on top of 2PACz, compared to 2PACz-only, facilitates more efficient hole transfer at the SAM/PM6 interface and suppresses trap-assisted recombination. These advantages are further supported by theoretical simulations that reveal enhanced orbital hybridization and interfacial charge-transfer pathways with the donor polymer PM6. Collectively, these effects lead to significantly improved device performance, with organic solar cells employing SC-coSAMu-based HTLs achieving a PCE of up to 20.1% with an FF exceeding 81%, outperforming 2PACz-only control devices (~ 18%). Moreover, this strategy is effective across multiple popular active-layer systems, delivering 7%−9% higher efficiencies and enhanced FFs in small-area (0.042 cm^2^) devices compared to using 2PACz alone. Importantly, the benefits translate from small-area cells to scale-relevant formats: a 17.14 cm^2^ mini-module (six serially connected subcells) reaches 17.0% efficiency, representing a substantial improvement over the 2PACz-based control. Overall, controlled multilayer co-assembly offers a general and scalable route to simultaneously optimize interfacial energetics and molecular organization, and should be extendable to other metal–oxide electrodes, active-layer systems, and optoelectronic devices where interfacial defects and large-area uniformity limit performance.

## Supplementary Information

Below is the link to the electronic supplementary material.

Supplementary file1 (PDF 3929 kb)
