# Supplementary material for: Layered Defect-Filling co-Assembled Carbazole-Based SAMs Deliver 20% Organic Solar Cells and 17% Mini-Modules
Source: Nanomicro Lett. 2026 Aug 10;19:29. doi: 10.1007/s40820-026-02325-2 (PMC13457534; doi:10.1007/s40820-026-02325-2)
Supplement: Supplementary file 1 [file 40820_2026_2325_MOESM1_ESM.pdf]

Supporting Information for

Layered Defect-Filling Co-Assembled Carbazole-Based SAMs Deliver 20% Organic Solar Cells and 17% Mini-Modules

Jingnan Wu[1,]*, Fengbo Sun[2], Leandro R. Franco[1], Qiaonan Chen[1,]*, Xinxin Xia[2], Ruike Zhou[3], Zhuang Li[3], Mateus Bergami[4], Pablo Hermosilla Fernandez[1], C. Moyses Araujo[4, 5], Biao Xiao[3], Xunchang Wang[3], Donghong Yu[6, 7], Maojie Zhang[2], Renqiang Yang[3,]*, Ergang Wang[1,]*

[1] Department of Chemistry and Chemical Engineering, Chalmers University of Technology, Göteborg, SE-412 96, Sweden

[2] National Engineering Research Center for Colloidal Materials, Key Laboratory of Special Functional Aggregated Materials (Shandong University), Ministry of Education, School of Chemistry & Chemical Engineering, Shandong University, Jinan, Shandong 250100, China.

[3] Key Laboratory of Flexible Optoelectronic Materials and Technology (Ministry of Education), School of Optoelectronic Materials & Technology, Jianghan University, Wuhan 430056, China

[4] Department of Engineering and Physics, Karlstad University, Karlstad 651 88, Sweden

[5] Materials Theory Division, Department of Physics and Astronomy, Uppsala University, Uppsala 751 20, Sweden

[6] Department of Chemistry and Bioscience, Aalborg University, DK-9220 Aalborg, Denmark

[7] Sino-Danish Center for Education and Research, DK-8000, Aarhus, Denmark

*Corresponding author. E-mail: jingnanw@chalmers.se (Jingnan Wu); qiaonan@chalmers.se (Qiaonan Chen); yangrq@jhun.edu.cn (Renqiang Yang); ergang@chalmers.se (Ergang Wang)

S1 Supplementary Text

S1.1. Materials

PM6 (Poly[[4,8-bis[5-(2-ethylhexyl)-4-fluoro-2-thienyl]benzo[1,2-b:4,5-b']dithiophene-2,6-diyl]-2,5-thiophenediyl[5,7-bis(2-ethylhexyl)-4,8-dioxo-4H,8H-benzo[1,2-c:4,5-c']dithiophene-1,3-diyl]-2,5-thiophenediyl]) was purchased from Solarmer Materials Inc..

L8-BO (2,2'-((2Z,2'Z)-((3,9-bis(2-butyloctyl)-12,13-bis(2-ethylhexyl)-12,13-dihydro-[1,2,5]thiadiazolo[3,4-e]thieno[2",3":4',5']thieno[2',3':4,5]pyrrolo[3,2-g]thieno[2',3':4,5]thieno[3,2-b]indole-2,10-diyl)bis(methanylylidene))bis(5,6-difluoro-3-oxo-2,3-dihydro-1H-indene-2,1-diylidene))dimalononitrile) was purchased from Solarmer Materials Inc..

PDINN (N,N'-bis{3-[3-(dimethylamino)propylamino]propyl}perylene-3,4,9,10-tetracarboxylic diimide) was purchased from Solarmer Materials Inc..

S1.2. Methods

The ultraviolet-visible (UV-vis) absorption spectra were recorded on a PerkinElmer lambda 1050 UV/vis/NIR spectrometer. Ultraviolet photoelectron spectroscopy (UPS) measurements were performed by a Thermo Fisher Scientific K-ALPHA+ system with He I (21.22 eV) as the excitation source. X-ray photoelectron spectroscopy (XPS) measurements were performed using a Thermo Scientific K-Alpha system. Atomic force microscopy (AFM) was performed through standard tapping-mode AFM measurements in ambient air on a scanned probe imaging and development (SPID) Park NX-10 imaging system. The AFM images were confirmed from

different samples and scan areas. The root-mean-square roughness (RMS) values of the height images were obtained from the entire scan area (2 × 2 μm) All AFM images were flattened and exported from the software (XEI). The current-voltage (*J-V*) characteristics were measured by a Keithley 2450 source measurement system. The OSCs were measured under an irradiation intensity of 100 mW/cm^2 (AM 1.5 G) using a Newport solar simulator, where the effective area of the device was 0.042 cm^2. The EQE spectra were analyzed by an integrated system (LST-QE), while the highly sensitive EQE was measured using an integrated system in which the photocurrent was amplified and modulated by a lock-in instrument.

S1.3. SCLC Mobility Measurement

The hole and electron mobilities of devices were evaluated from triplicates from a same device via space-charge-limited current (SCLC) method with hole-only structure of ITO/HTL/Active layer/MoO_3/Ag and electron-only structure of ITO/ZnO/ Active layer/PDINN/Ag, respectively. The corresponding charge mobilities were calculated via fitting Mott-Gurney square law $J = 9\varepsilon_r\varepsilon_0\mu V^2/(8L^3)$, whereas J is the current density, ε_r is the dielectric permittivity of the active layer (assumed to be 3), ε_0 is the vacuum permittivity, L is the thickness of the active layer, μ is the hole or electron mobility. $V = V_{appl} - V_{bi} - V_s$, V_{appl} is the applied voltage, V_{bi} is the built-in voltage, and V_s is the voltage drop from the substrate's series resistance (V_s = IR). The SCLC devices were measured under a dark condition in a nitrogen glovebox without encapsulation.

S1.4. Contact angle measurement

Contact angles of distinct solutions (deionized water and diiodomethane) on hole transport layer films were measured by using Dataphysics-OCA20 Micro surface contact angle analyzer.

S1.5. GIWAXS measurement

GIWAXS characterization was conducted by 2D-GIWAXS experiments using a GANESHA 300XL+ system from JJ X-ray. The instrument is equipped with a Pilatus 300K detector with a pixel size of 172 × 172 μm. The X-ray source is a Genix 3D Microfocus sealed tube X-ray Cu-source with an integrated monochromator (30 W). The wavelength used was Q = 1.5418 Å. The detector moved in a vacuum chamber with a sample-to-detector distance varied between 0.115 m and 1.47 m, depending on the configuration, as calibrated by silver behenate (d001 = 58.380 Å). The minimized background scattering plus high-performance detector allowed for a detectable q-range varying from 3×10^{-4} to 3 Å^{-1} (0.2 to 210 nm). The sample was placed vertically on the goniometer and tilted to a glancing angle of 0.2° with respect to the incoming beam. A small beam was then used to obtain better resolution. The accumulation time was 30 min for each measurement. In-plane and out-of-plane line-cuts were obtained using the SAXSGUI program.

S1.6. TPC and TPV measurement

Transient photovoltage (TPV) and transient photocurrent (TPC) measurements were carried out under a 337 nm 3.5 ns pulse laser (160 μJ per pulse at 10 Hz) and halide lamps (150 W). Voltage and current dynamics were recorded on a digital oscilloscope (Tektronix MDO3102).

S1.7. Theoretical Modelling

The ground and excited states electronic structures of 2PACz, 2Cl-4PACz and PM6 were initially investigated by employing standard quantum chemistry calculations using Density Functional Theory (DFT) and Time-Dependent DFT (TD-DFT) methods. Properties of interest, as frontiers molecular orbitals energy and density distribution and excited states energy, intensity and character were calculated at the ground state optimized geometry in gas phase. Calculations were performed using the B3LYP [1] exchange-correlation function combined with the triple-zeta quality basis set 6-311G(d,p) [2], as implemented in the Gaussian 16

program (Rev C.01) [3]. We investigated both isolated molecules and interfaces formed between self-assembled monolayers (SAMs) and PM6 to gain insights into their structural and electronic properties. In these models and also as an isolated system, PM6 was represented in our calculations as a single monomer, with methyl groups replacing the longer side chains to reduce computational cost while retaining the key electronic features of the polymer. Given the complexity of the configurational space associated with such interfaces, we constructed six distinct configurations by systematically varying the relative orientation between the SAM and PM6. This approach allowed us to sample different local minima on the potential energy hypersurface, providing a more comprehensive exploration of the system's stability and electronic structure. To identify the most favorable configurations, we performed geometry optimizations at the DFT level, accounting for intermolecular interactions and structural relaxation effects. The relative stability of each configuration was assessed based on total energy calculations, while key electronic properties —such as excited states charge transfer characteristics, molecular orbital alignment, and interfacial electronic coupling— were analyzed to determine their impact on charge transport and excitonic processes. The one-electron transition density matrix (^{1}TDM) [4] together with the natural transition orbitals were calculated to characterize the nature of the excited states.

Solid state DFT calculations were performed to investigate the electronic structure of ITO with absorbed 2PACz, 2Cl-4PACz molecules. The ITO was modeled using a slab with I60Sn4O96 stoichiometry, featuring approximately 27 Å of vacuum along the z-axis. The molecules were adsorbed on only one side of the slab. We constructed two models: one in which a 2PACz molecule interacts with another 2PACz, and another where a 2Cl-4PACz molecule interacts with a 2PACz. In spin-coated films with SAMs on ITO, the SAM molecules tend to form a complex multilayer structure, posing a challenge for *ab initio* calculations. To model the adsorption and interaction of these SAMs on ITO, we initially adsorbed a 2PACz molecule in a tridentate configuration, known as the minimum energy state [5, 6]. A second molecule (either 2PACz or 2Cl-4PACz) was positioned near the first, approximately 1 Å from the carbazole units and 1 Å from the surface. These configurations were fully relaxed as described below.

In order to solve the Kohn-Sham equations, we employed the Perdew-Burke-Ernzerhof (PBE) generalized gradient approximation [7] alongside the all-electron projector augmented-wave (PAW) method [8, 9]. The equilibrium volumes for the I60Sn4O96 slabs, both with and without adsorbed molecules, were determined by optimizing the stress tensor and atomic forces using a plane-wave cutoff energy of 500 eV and integrating over the Brillouin zone with a 5×5×1 k-mesh. Equilibrium crystal structures were achieved once the atomic forces on all atoms fell below 0.010 eV/Å and the total energy converged to within 10^{-5} eV. For the work function calculations, we enhanced the precision by employing a 6×6×1 k-mesh. Dispersion effects were incorporated using the Becke-Johnson (BJ) damping variant of the DFT-D3 van der Waals correction [10]. All calculations were performed with version 5.4.1 of the Vienna Ab Initio Simulation Package (VASP) [11, 12].

To investigate the solution-phase aggregation behavior of the SAMs molecules, classical molecular dynamics (MD) simulations were performed using the GROMACS package [13]. Force field parameters were assigned according to the OPLS/AA framework [14-16], and atomic partial charges for 2PACz and 2Cl-4PACz were derived using the CHELPG scheme at the B3LYP/6-311G(d,p) level, including implicit solvation via the polarizable continuum model (PCM) for the solvent ethanol as implemented in the Gaussian 16 program (Rev C.01) [4]. Three simulation systems were constructed to reproduce experimentally relevant concentrations of SAMs (15 mg mL^{-1}): (i) 2PACz solvated in ethanol, (ii) 2Cl-4PACz solvated in ethanol, and (iii) a mixed system containing a 1:1 molar ratio of 2PACz and 2Cl-4PACz under identical conditions. Approximately 5000 ethanol molecules were considered. Each

system was equilibrated and subsequently propagated for 100 ns in the NPT ensemble at T = 298.15 K and p = 1 atm. Temperature and pressure were controlled using the V-rescale thermostat and C-rescale barostat, respectively. Intermolecular interactions were quantified by monitoring close contacts between SAMs molecules, defined as any interatomic separation shorter than 4 Å. Time-resolved contact analysis revealed that pure 2PACz and pure 2Cl-4PACz tend to self-aggregate in ethanol. In contrast, in the 1:1 mixed system, 2PACz - 2Cl-4PACz heteromolecular contacts occurred at a significantly higher frequency than interactions between identical molecules. These results indicate an enhanced propensity for heteromolecular aggregation in ethanol, supporting a solution-phase pre-organization mechanism that should drive the formation of the multilayers on top of ITO electrode.

S2 Supporting Figures

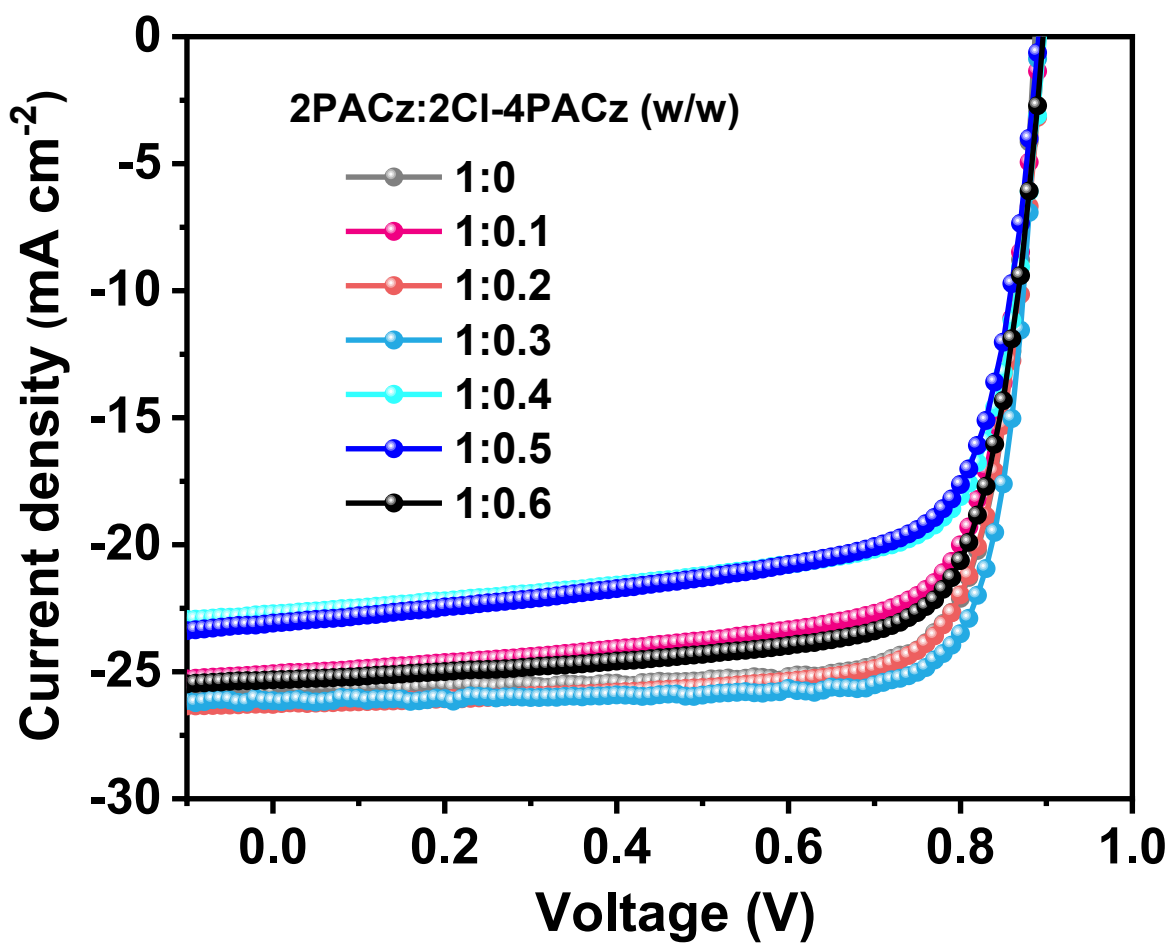

Fig. S1. The *J-V* characteristics of the OSCs based on PM6:L8-BO with different BC-coSAMu compositions (2PACz:2Cl-4PACz = 1:X, w/w) as HTLs under the irradiation of AM 1.5G, 100 mW/cm^2.

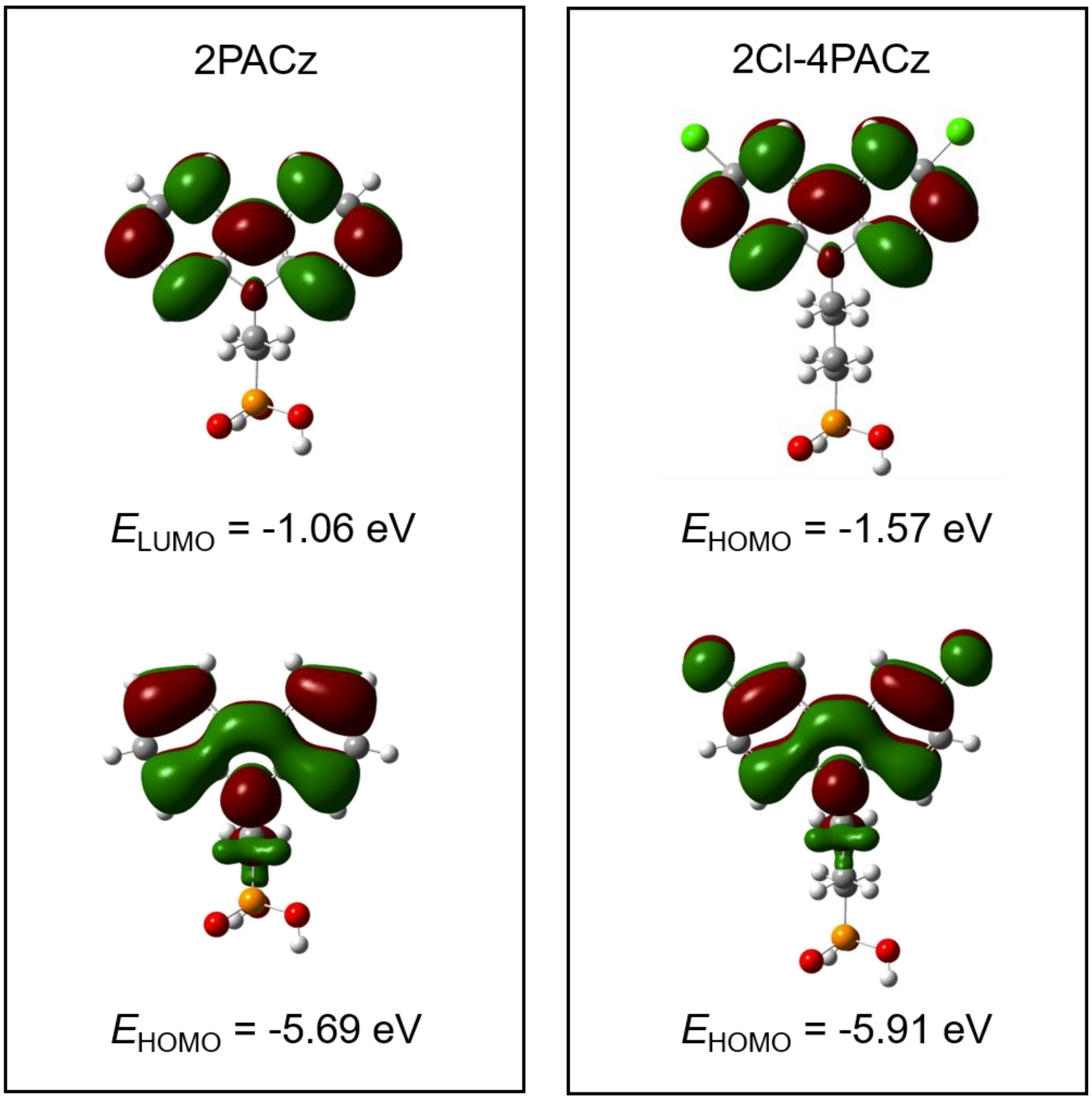

Fig. S2. HOMO and LUMO density distributions of 2PACz and 2Cl-4PACz.

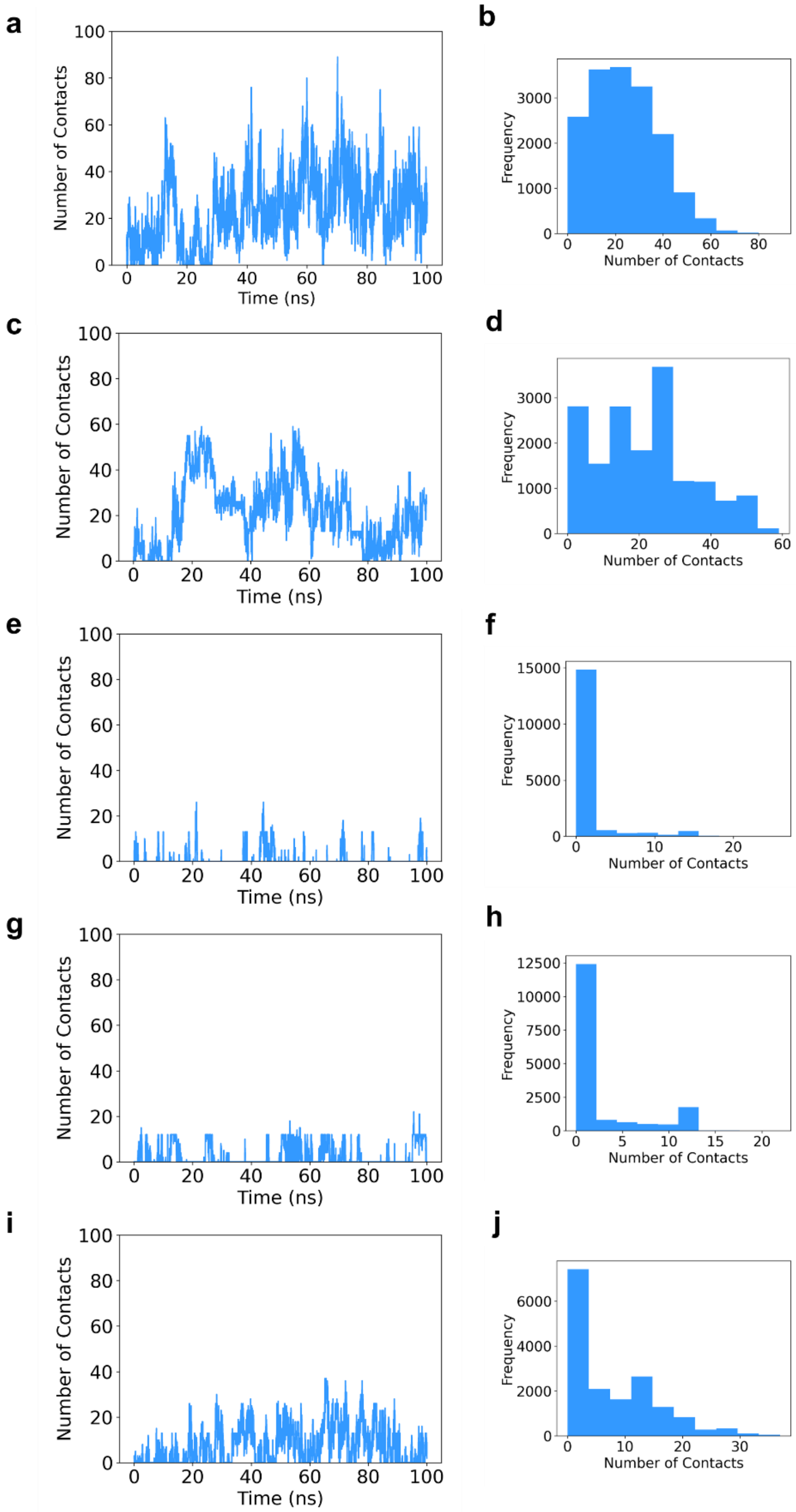

Fig. S3. a,b,e,f,g,h Time evolution of the number of intermolecular close contacts and histogram distributions of the mixture 1:1. **a,b** Heteromolecular 2PACz_2Cl-4PACz. **e,f** Homomolecular 2PACz_2PACz. **g,h** Homomolecular 2Cl-4PACz_2Cl-4PACz. **c,d,i,j** Time evolution of the number of intermolecular close contacts and histogram distributions of the 2Cl-4PACz solution in ethanol and the 2PACz solution in ethanol. (c,d) Homomolecular 2Cl-4PACz_2Cl-4PACz, time evolution of the number of intermolecular close contacts, and histogram distributions of 2Cl-4PACz solution in ethanol. (i,j) Homomolecular 2PACz_2PACz,

time evolution of the number of intermolecular close contacts, and histogram distributions of 2PACz solution in ethanol.

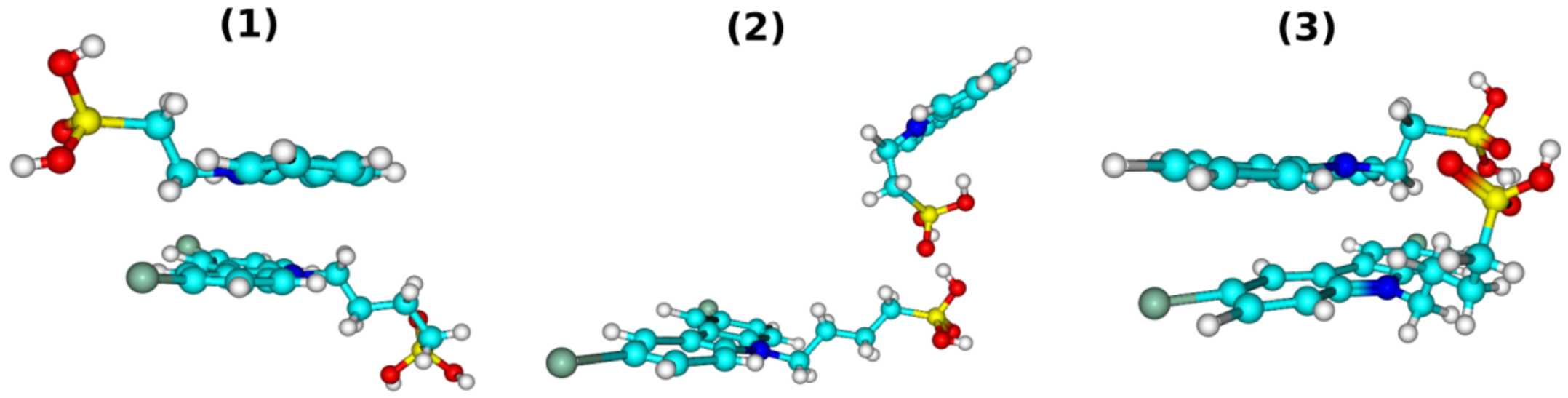

Fig. S4. Representative dimeric structures extracted from the 1:1 mixed 2PACz/2Cl-4PACz MD simulation, illustrating the main intermolecular interaction motifs that drive association in ethanol. The dominant configurations include: (1) π-π stacking between the carbazole rings of 2PACz and 2Cl-4PACz, with opposed anchoring groups; (2) close contacts resembling hydrogen bonds between oxygen and hydrogen atoms of the anchoring groups; and (3) dimers in which both π-π stacking and hydrogen bonds occur simultaneously.

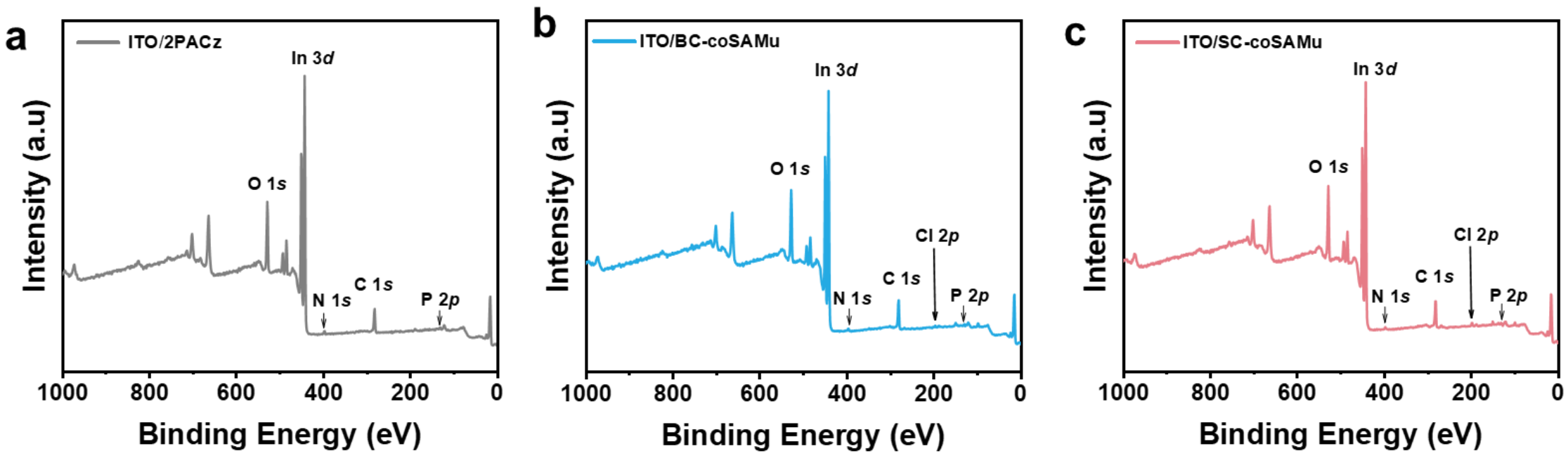

Fig. S5. XPS surveys of 2PACz-, BC-coSAMu-, and SC-coSAMu-modified ITO substrates.

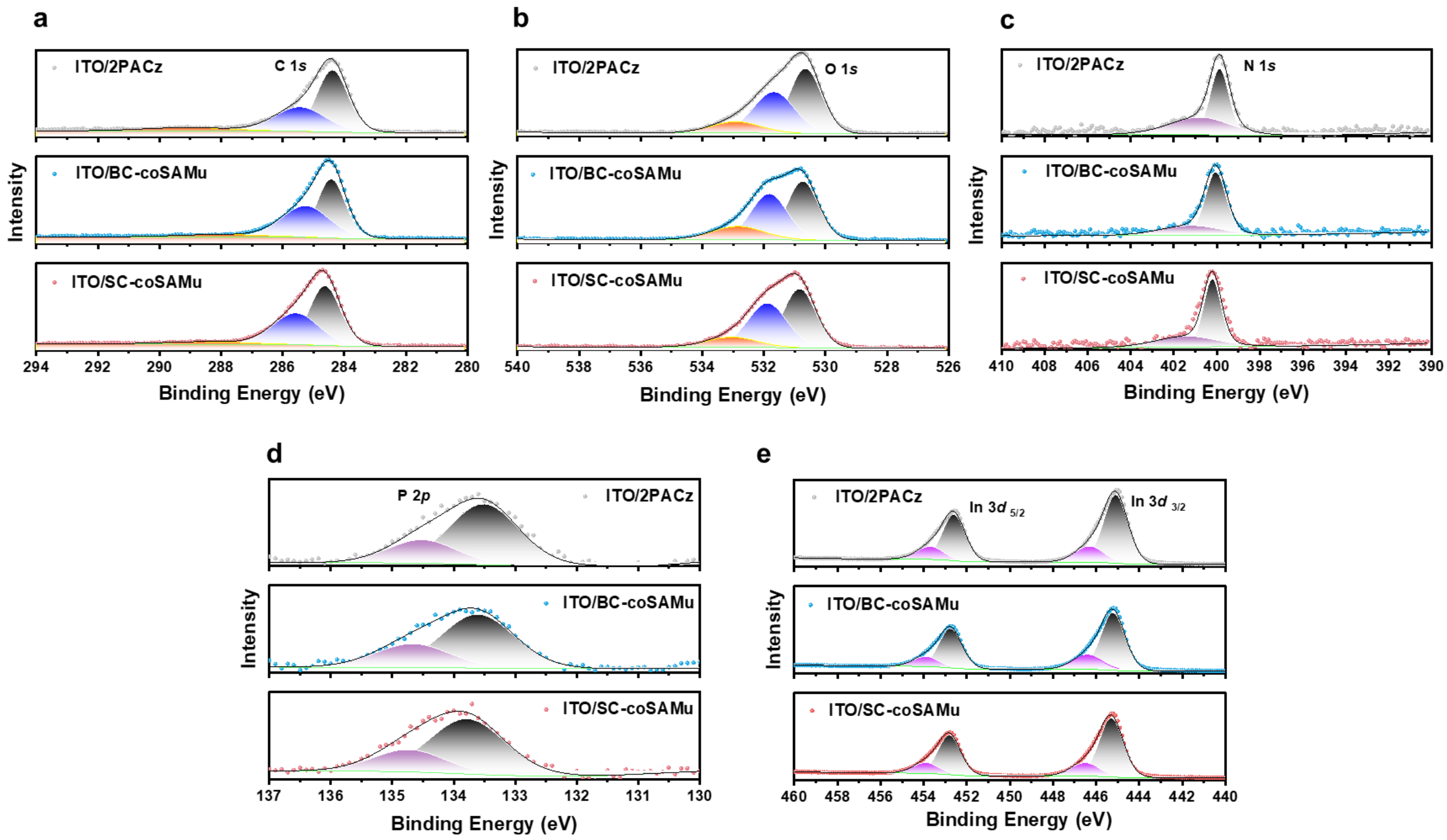

Fig. S6. XPS analysis of the **a** C 1*s*, **b** O 1*s*, **c** N 1*s*, **d** P 2*p* and (e) In 3$d_{3/2}$ core-level regions for 2PACz-, BC-coSAMu-, and SC-coSAMu-modified ITO substrates.

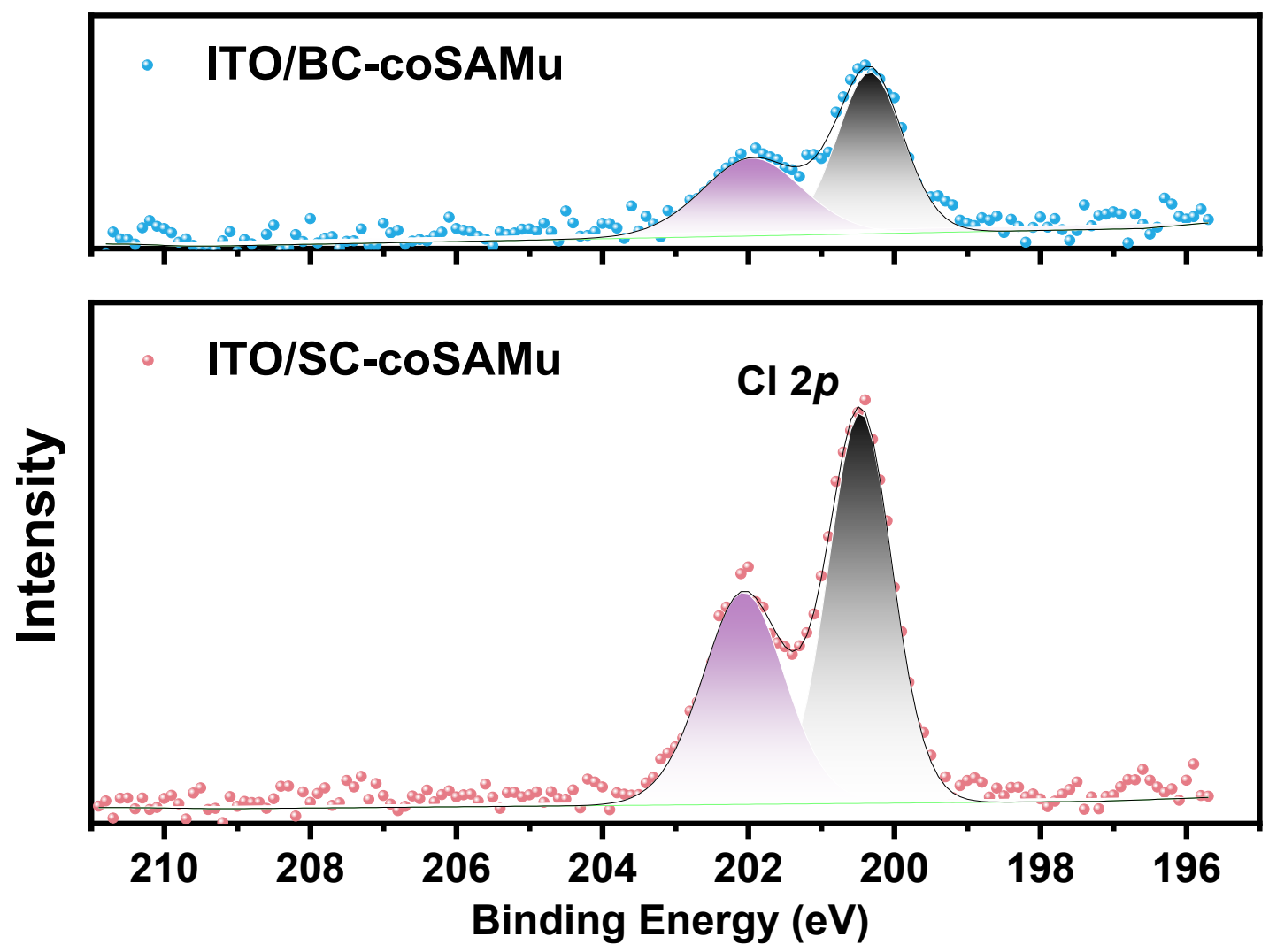

Fig. S7. XPS analysis of the Cl 2*p* core-level regions for BC-coSAMu- and SC-coSAMu-modified ITO substrates.

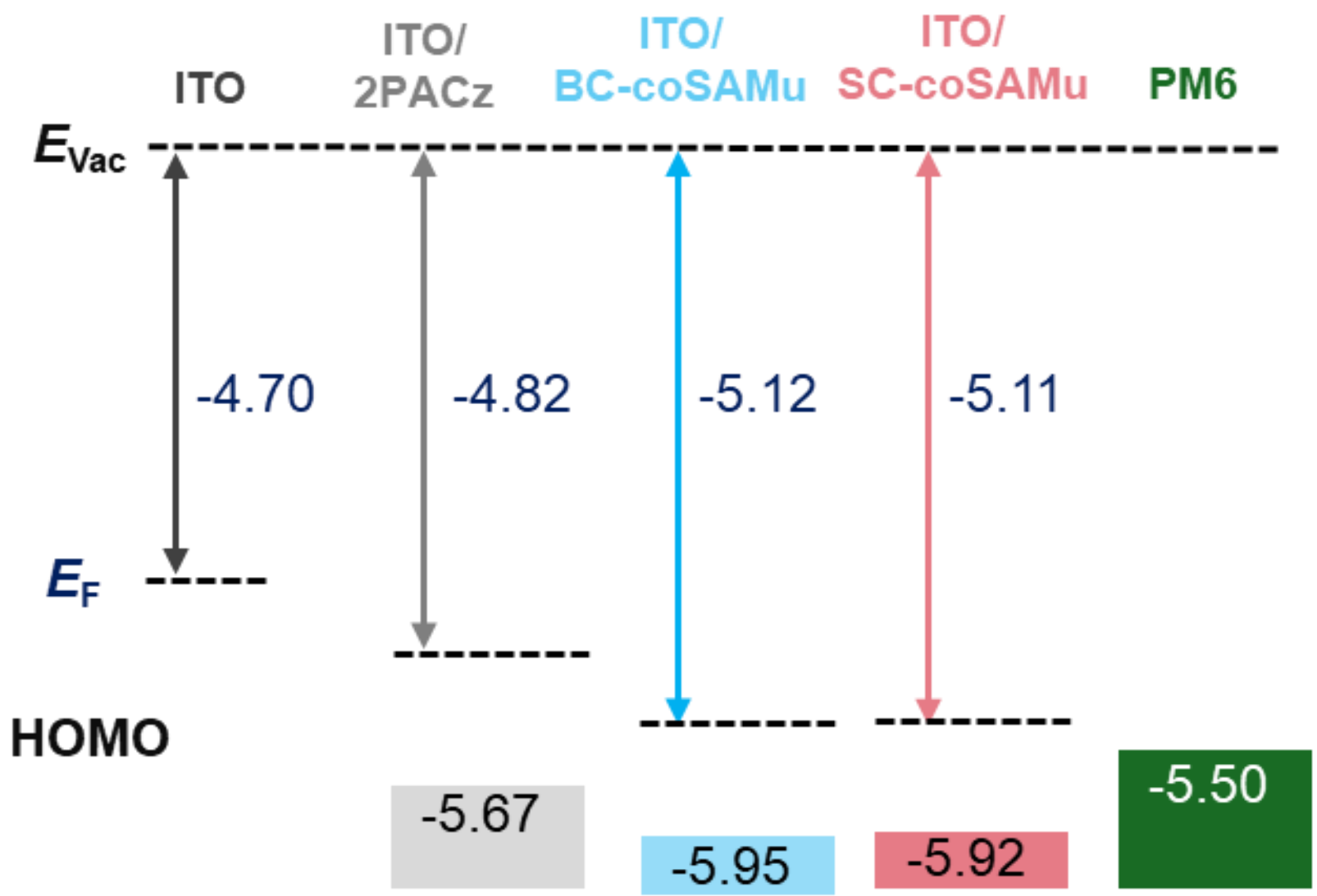

Fig. S8. UPS-measured energy levels of ITO, SAM modified ITOs, together with the HOMO levels of PM6 [17].

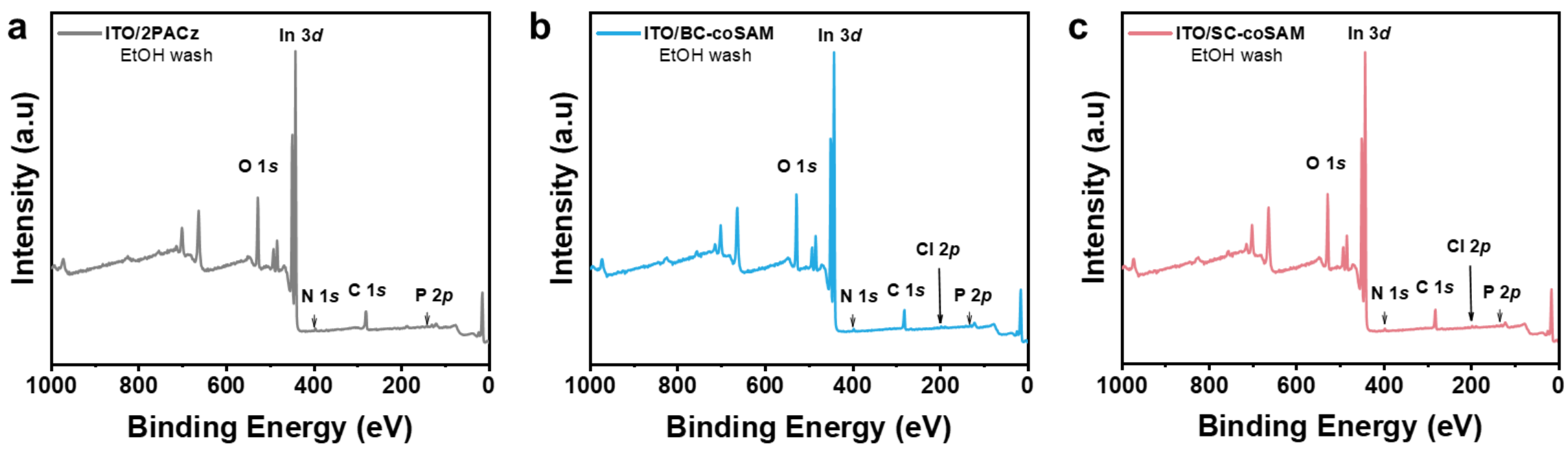

Fig. S9. XPS surveys of 2PACz, BC-coSAMu- and SC-coSAMu-modified ITO substrates with two times ethanol wash.

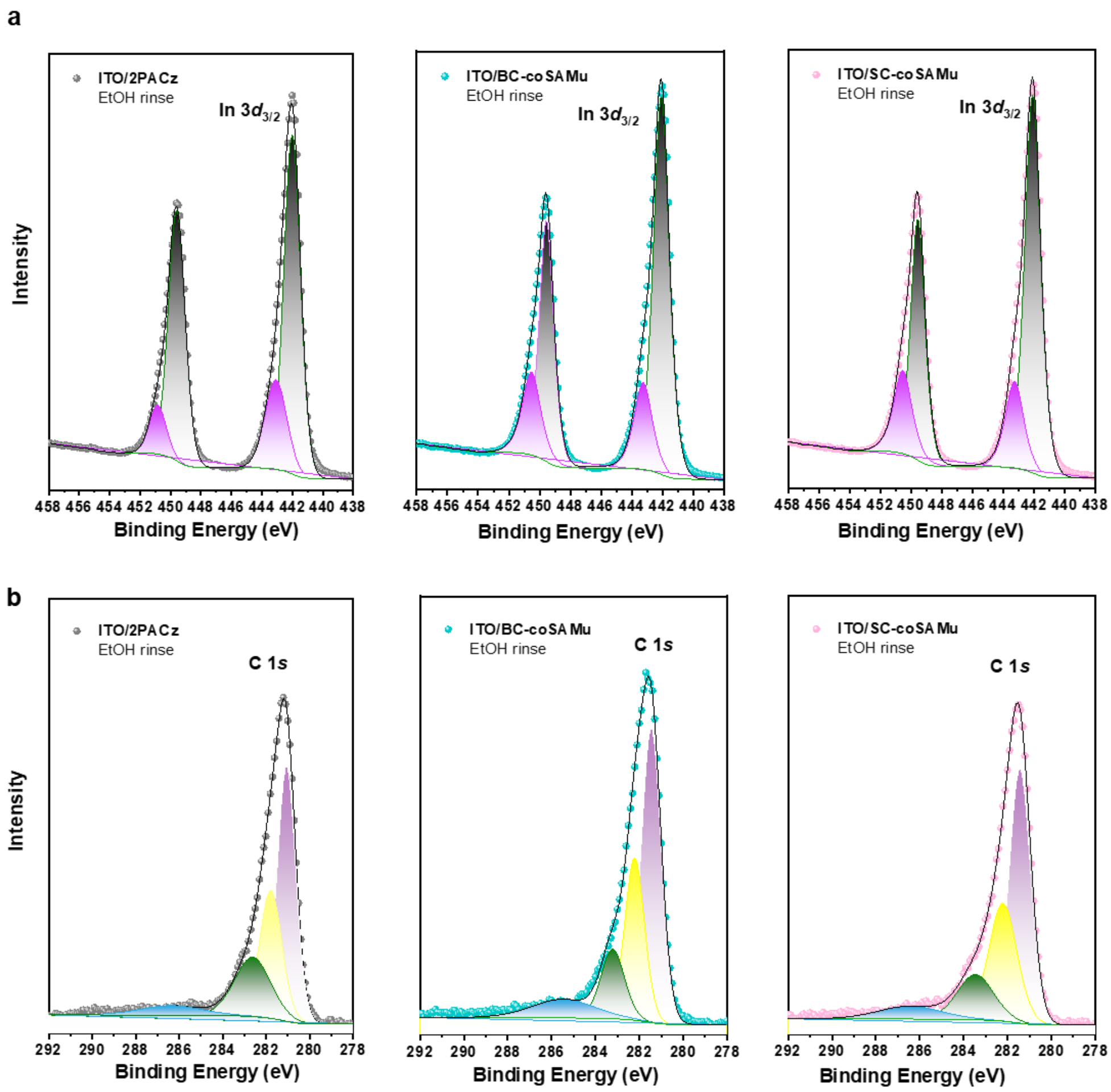

Fig. S10. XPS analysis of the C 1s, Cl 2p and In 3$d_{3/2}$ core-level regions for 2PACz, BC-coSAMu- and SC-coSAMu-modified ITO substrates with two times ethanol rinse.

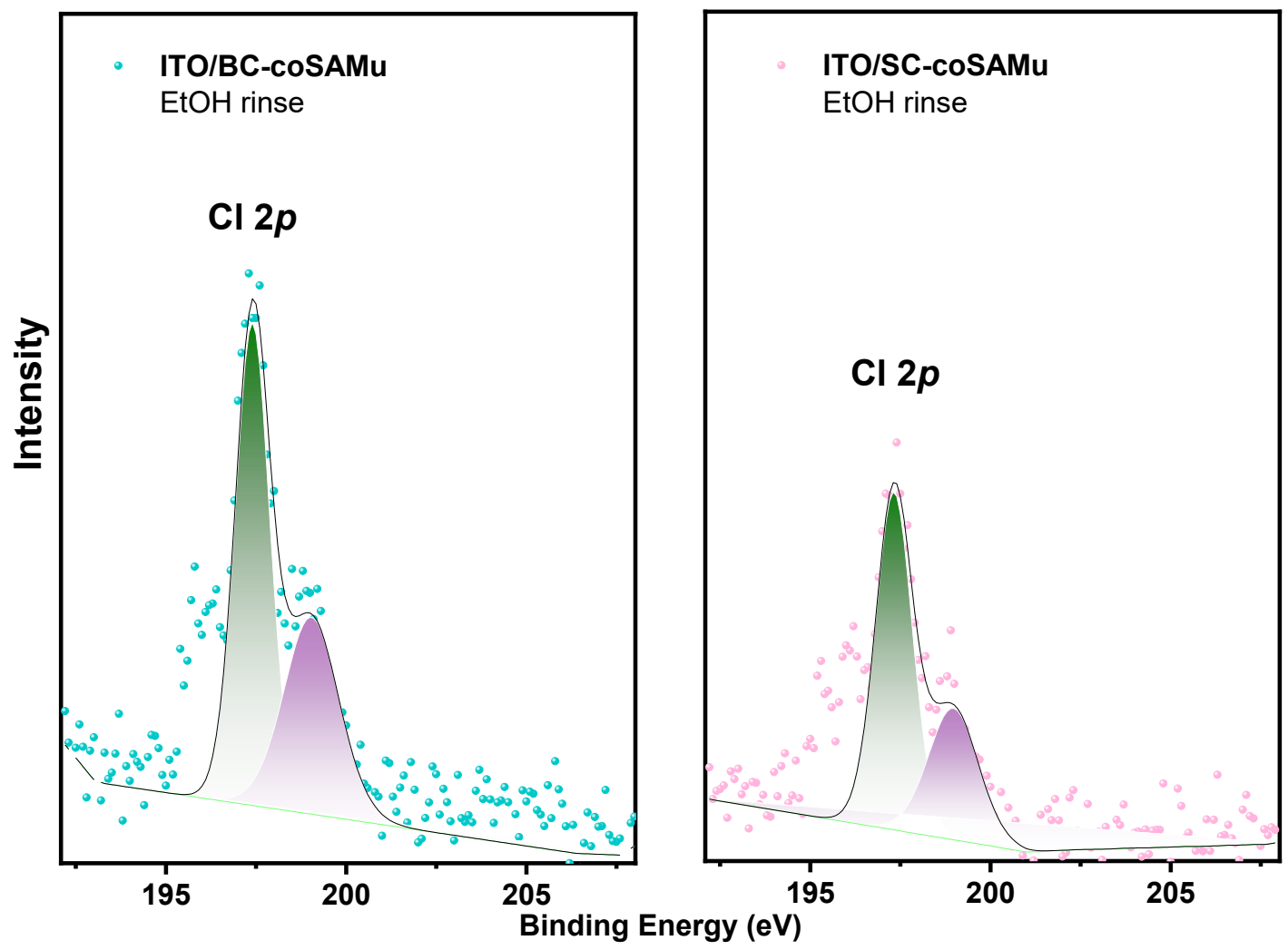

Fig. S11. XPS analysis of the Cl 2p core-level regions for BC-coSAMu- and SC-coSAMu-modified ITO substrates with two times ethanol rinse.

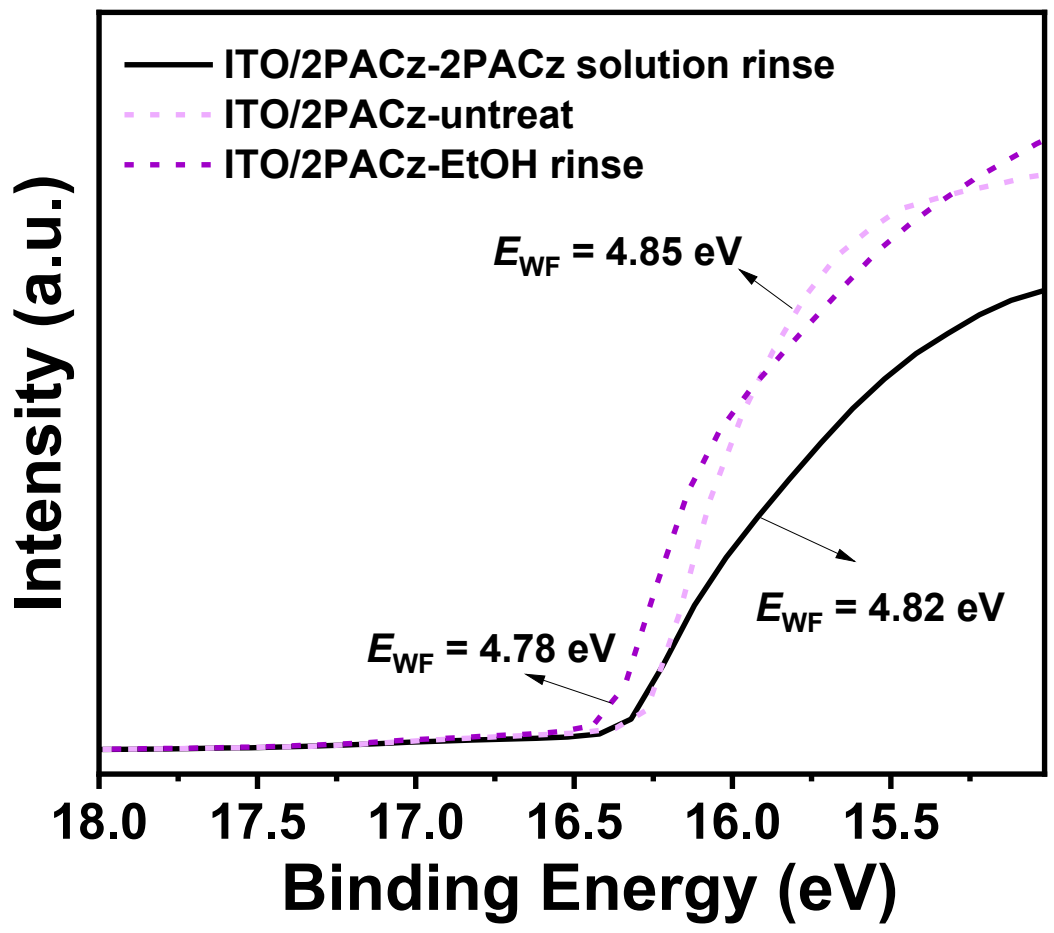

Fig. S12. UPS spectra (He I lamp with the photon energy of 21.22 eV) of 2PACz-modified ITO for direct determination of the WF.

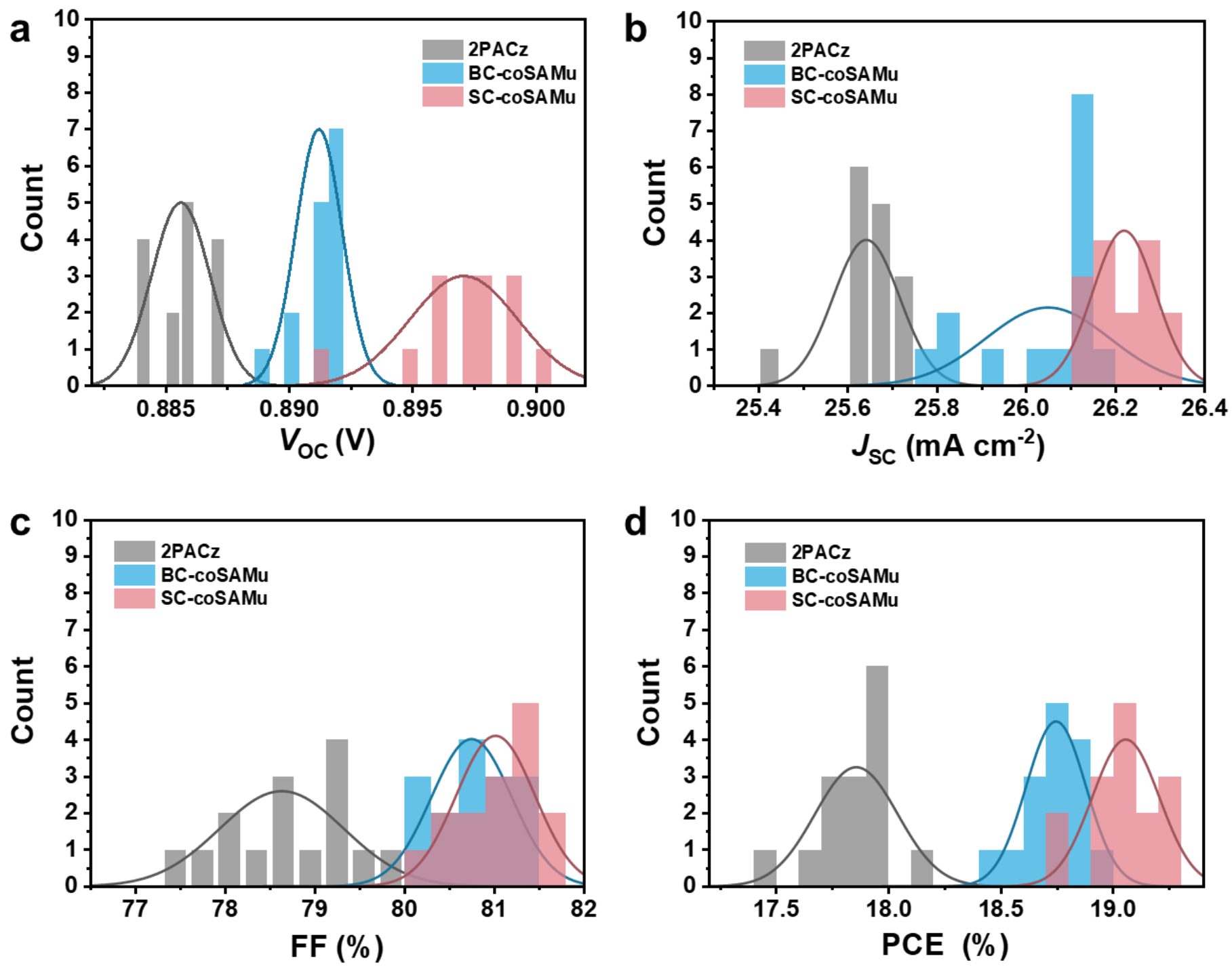

Fig. S13. The histogram of **a** V_{OC}, **b** J_{SC}, **c** FF, and **d** PCE of PM6:L8-BO-based OSCs.

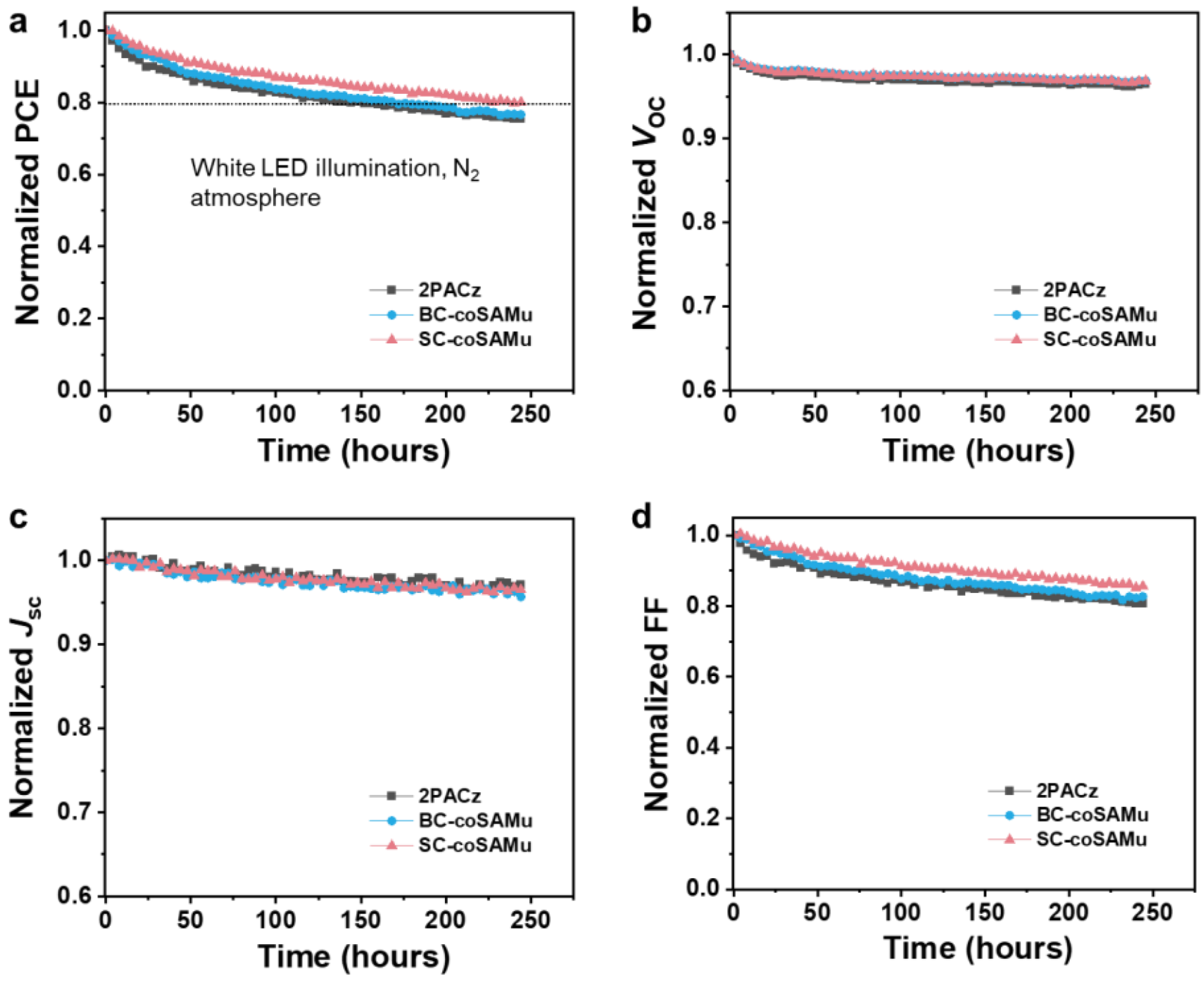

Fig. S14. Normalized **a** PCE, **b** V_{OC}, **c** J_{SC}, **d** FF of the PM6:L8-BO-based devices with different HTLs under continuous white LED illumination at the maximum power point in nitrogen.

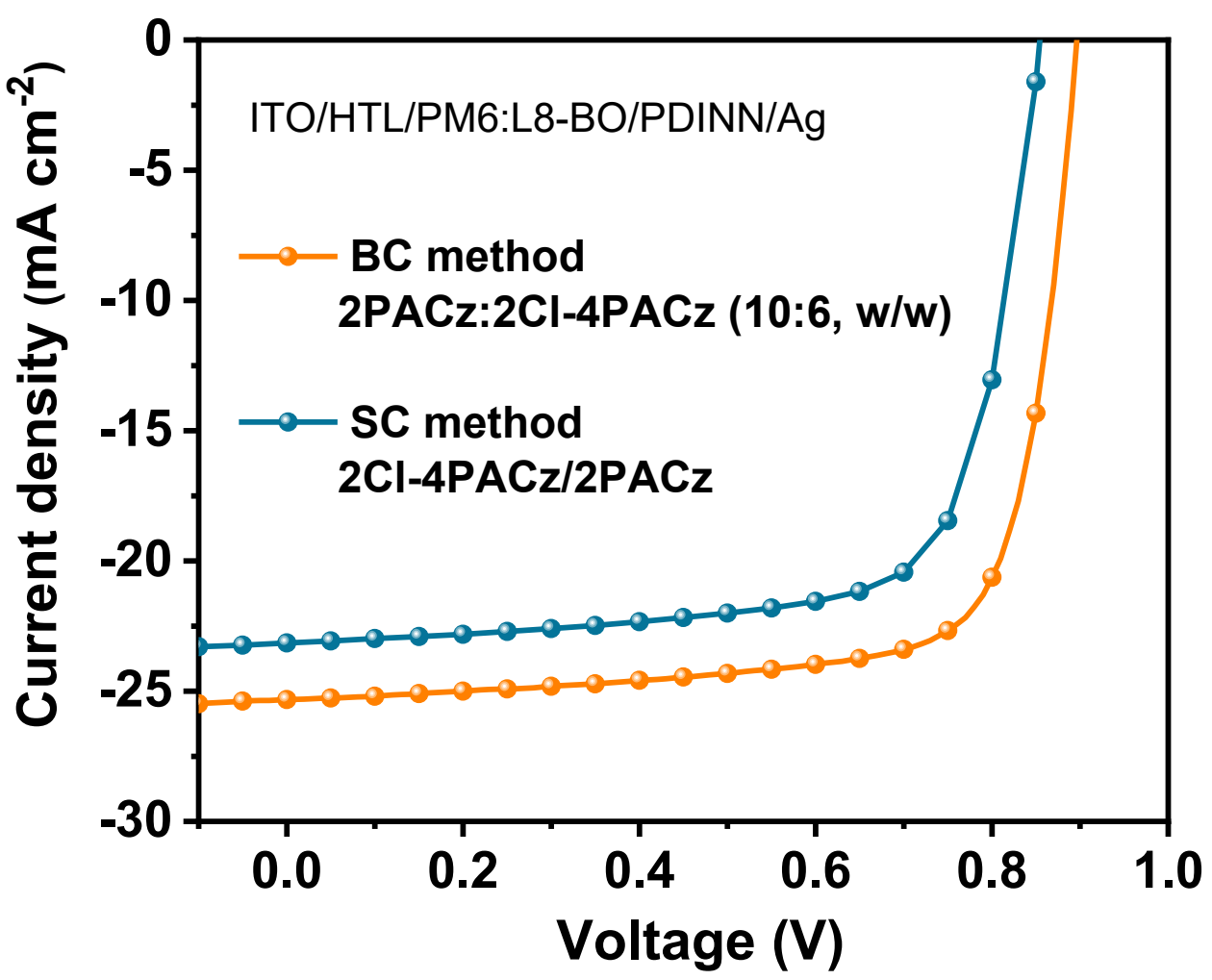

Fig. S15. The J–V characteristics of PM6:L8-BO solar cells incorporating different HTLs: 2PACz:2Cl-4PACz (10:6, w/w) and sequentially deposited 2Cl-4PACz/2PACz under the irradiation of AM 1.5G, 100 mW/cm^2.

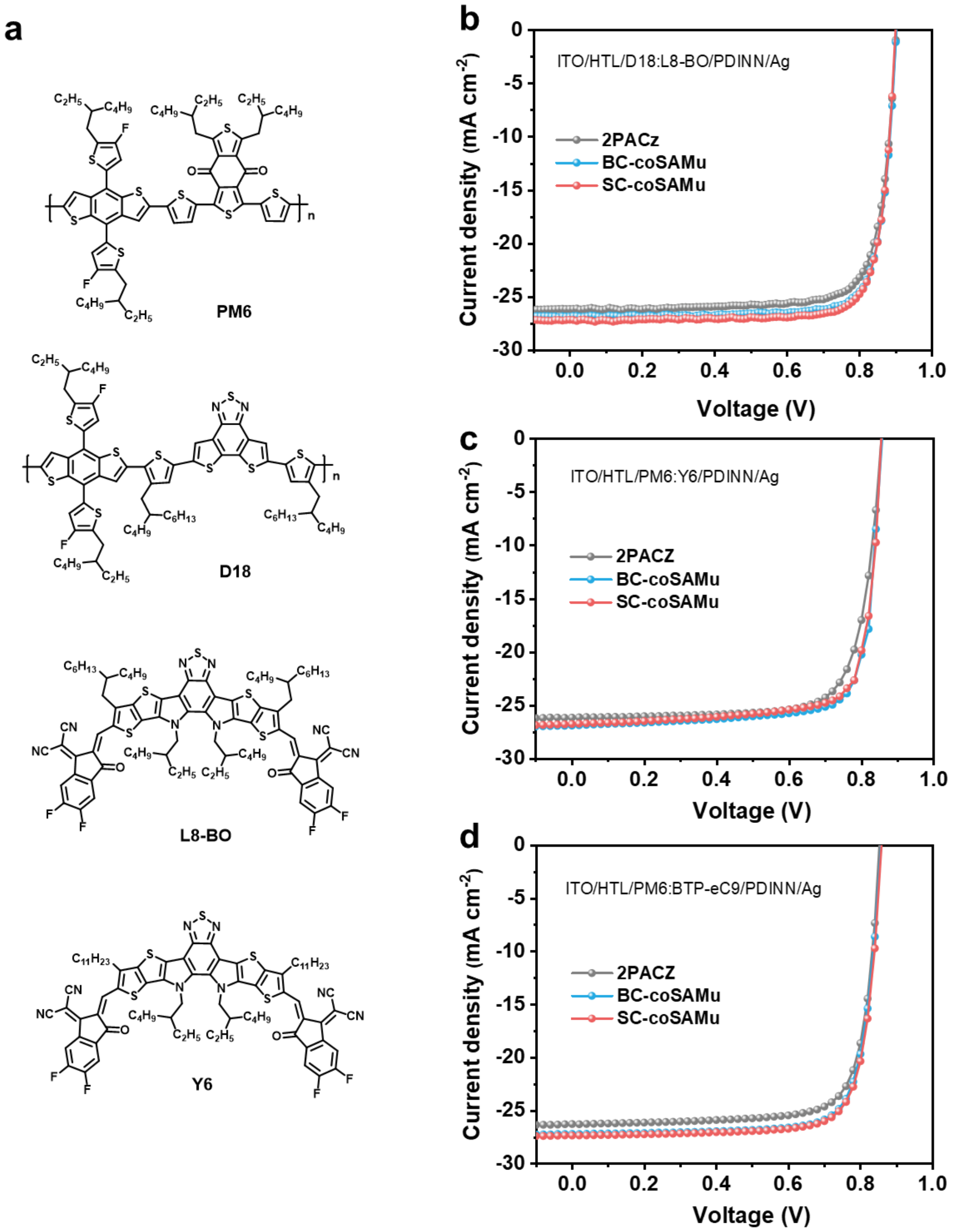

Fig. S16. a Molecular structures of PM6, L8-BO, D18 and Y6. The *J-V* characteristics of the OSCs based on **b** D18:L8-BO, **c** PM6:Y6 and **d** PM6:BTP-eC9, with different HTLs under the irradiation of AM 1.5G, 100 mW/cm^2.

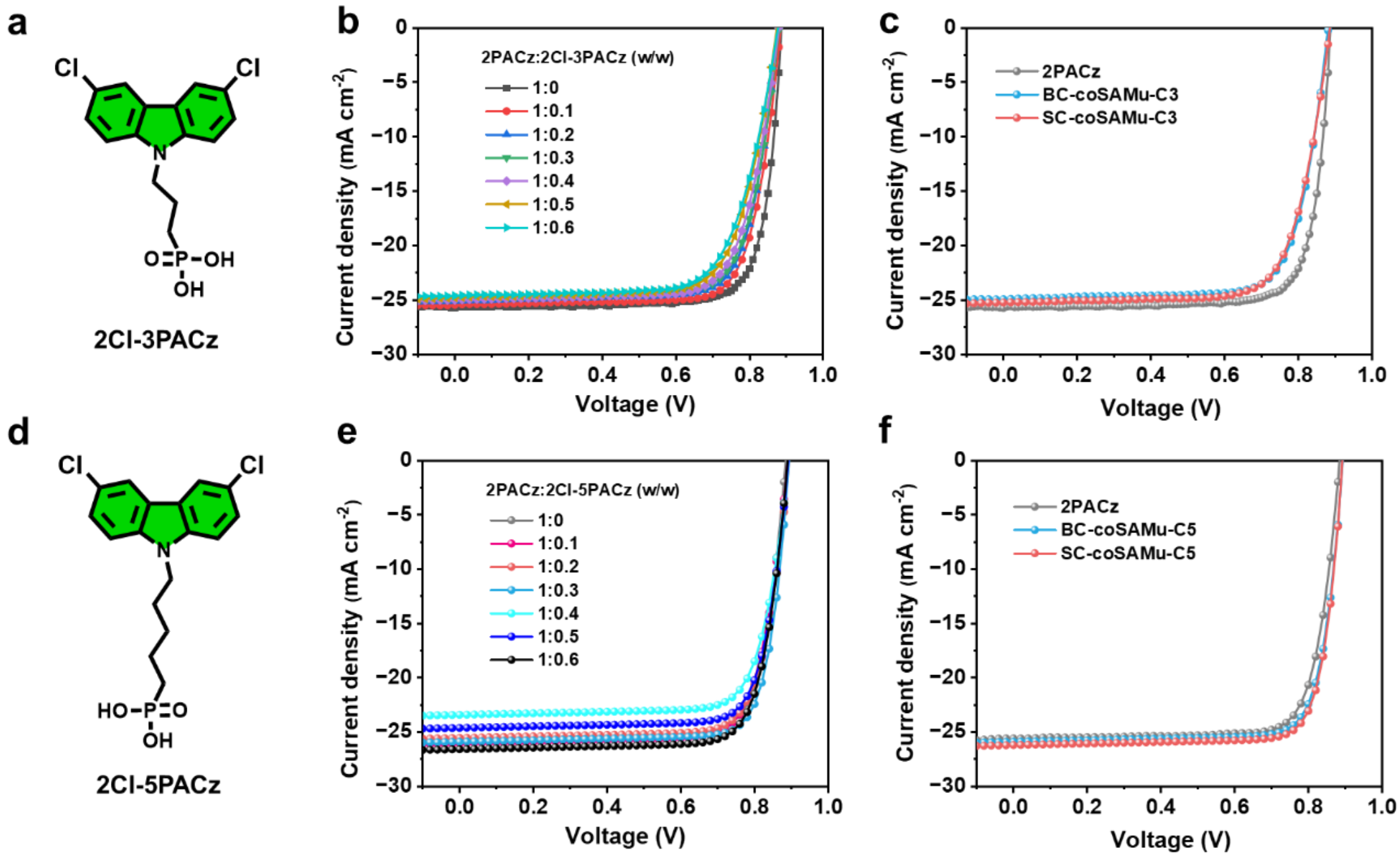

Fig. S17. Chemical structures of **a** 2Cl-3PACz and **d** 2Cl-5PACz. *J–V* characteristics of PM6:L8-BO-based organic solar cells employing BC-coSAMu hole-transport layers with varying compositions of **b** 2PACz:2Cl-3PACz = 1:X (w/w) and **e** 2PACz:2Cl-5PACz = 1:X (w/w). *J–V* characteristics of devices with different HTLs based on **c** 2Cl-3PACz and **f** 2Cl-5PACz systems. All measurements were performed under AM 1.5G illumination (100 mW cm^{-2}).

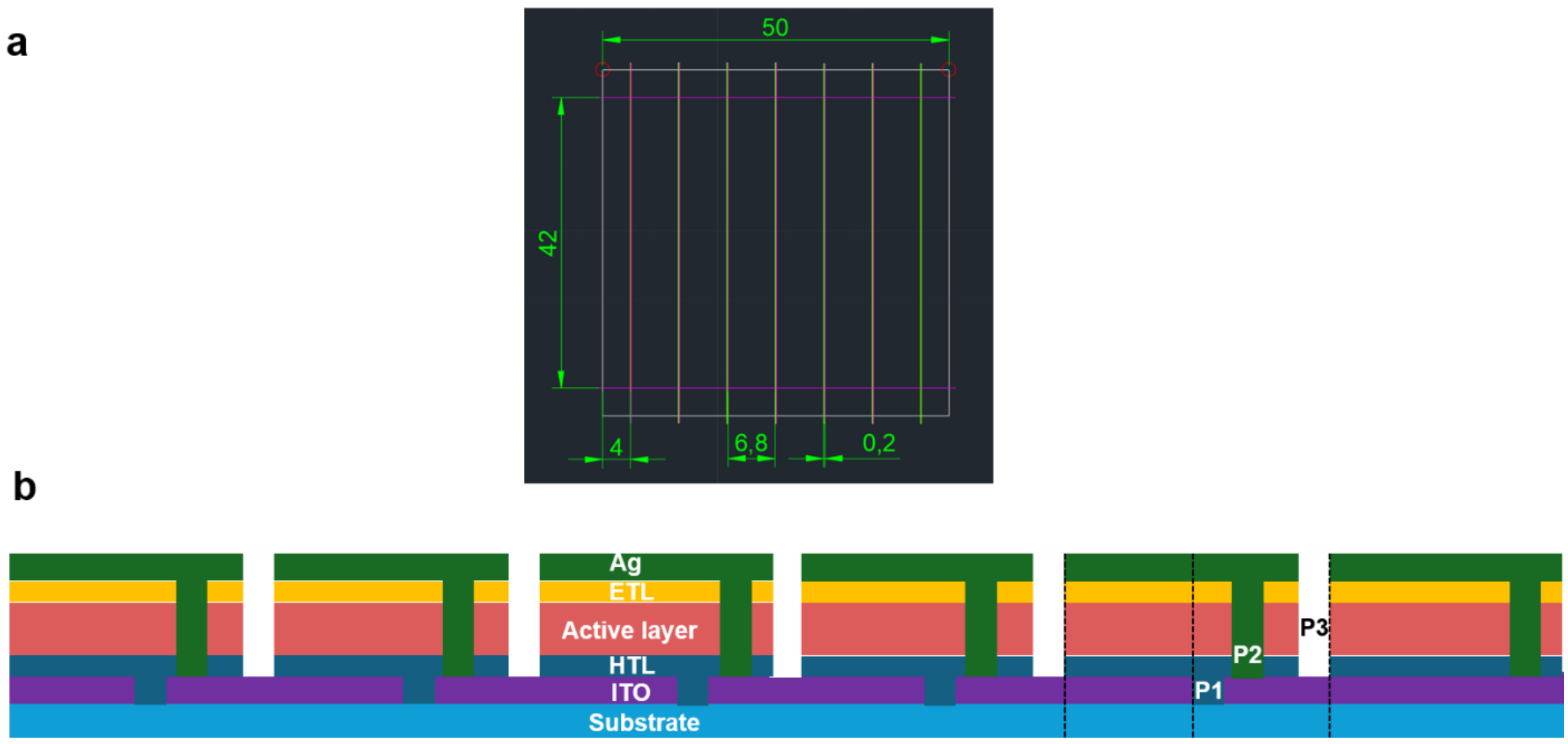

Fig. S18. a Schematic of the module configuration. **b** Schematic of a large-area OSC module comprising six individual cells connected in series.

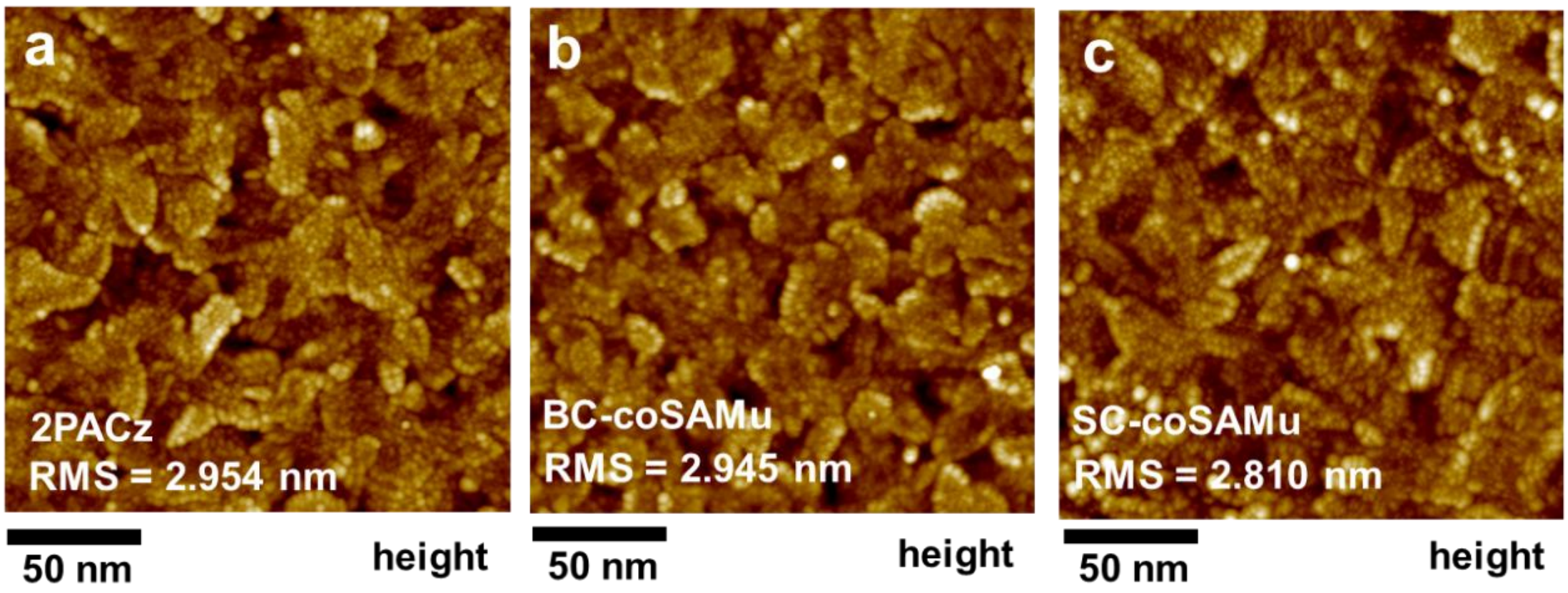

Fig. S19. Tapping-mode AFM height images of 2PACz, BC, and SC-coSAMu films on ITO substrates, respectively.

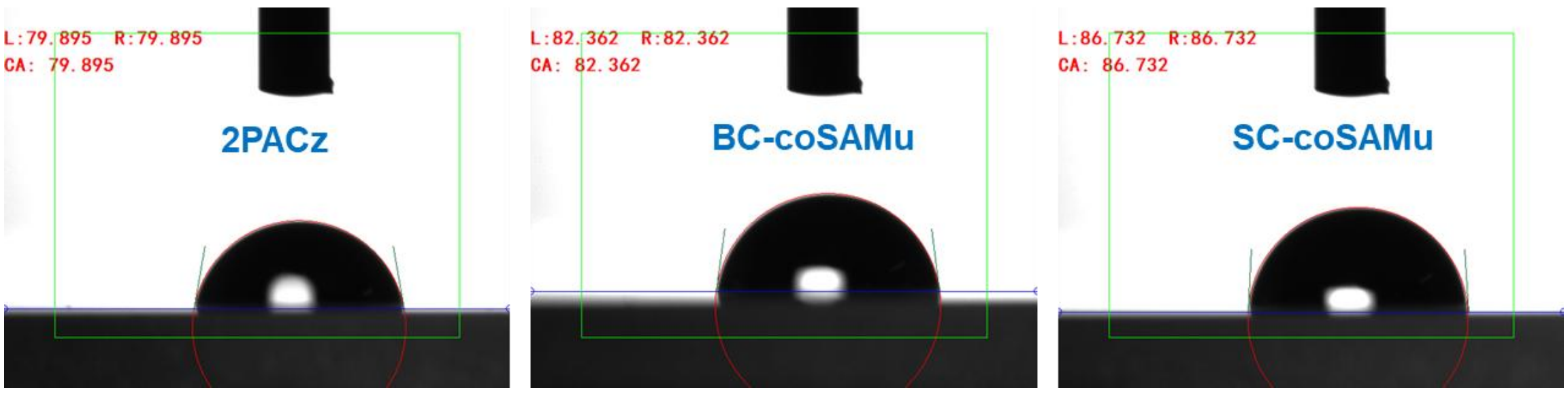

Fig. S20. The contact angle images of 2PACz, BC-coSAMu- and SC-coSAMu films performed by using deionized water (H_2O) as wetting liquids.

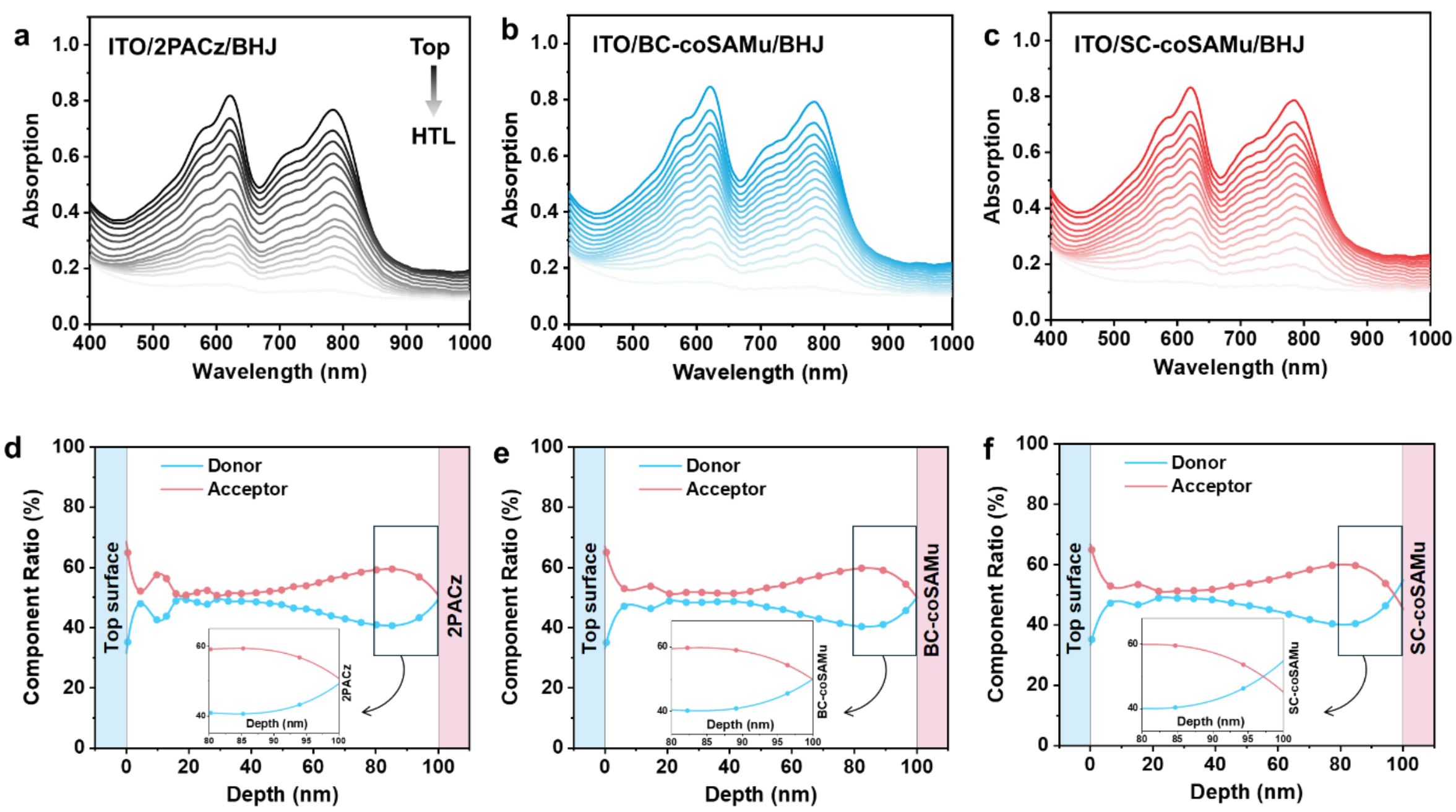

Fig. S21. a-c Film-depth-dependent absorption spectra and **d-f** calculated component distribution profiles of the PM6:L8-BO blended film on 2PACz, BC-coSAMu- and SC-

coSAMu films as HTLs.

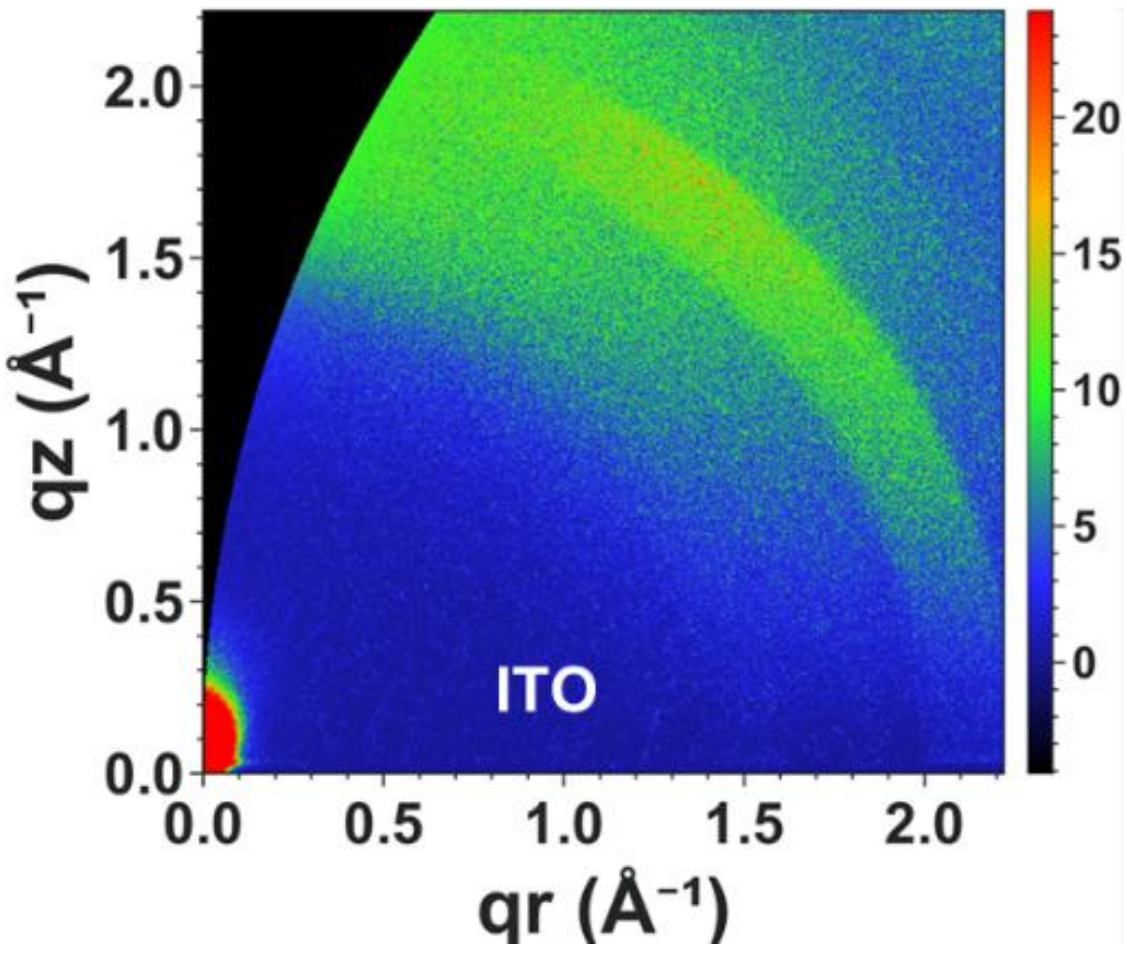

Fig. S22. 2D GIWAXS patterns of ITO glass substrates.

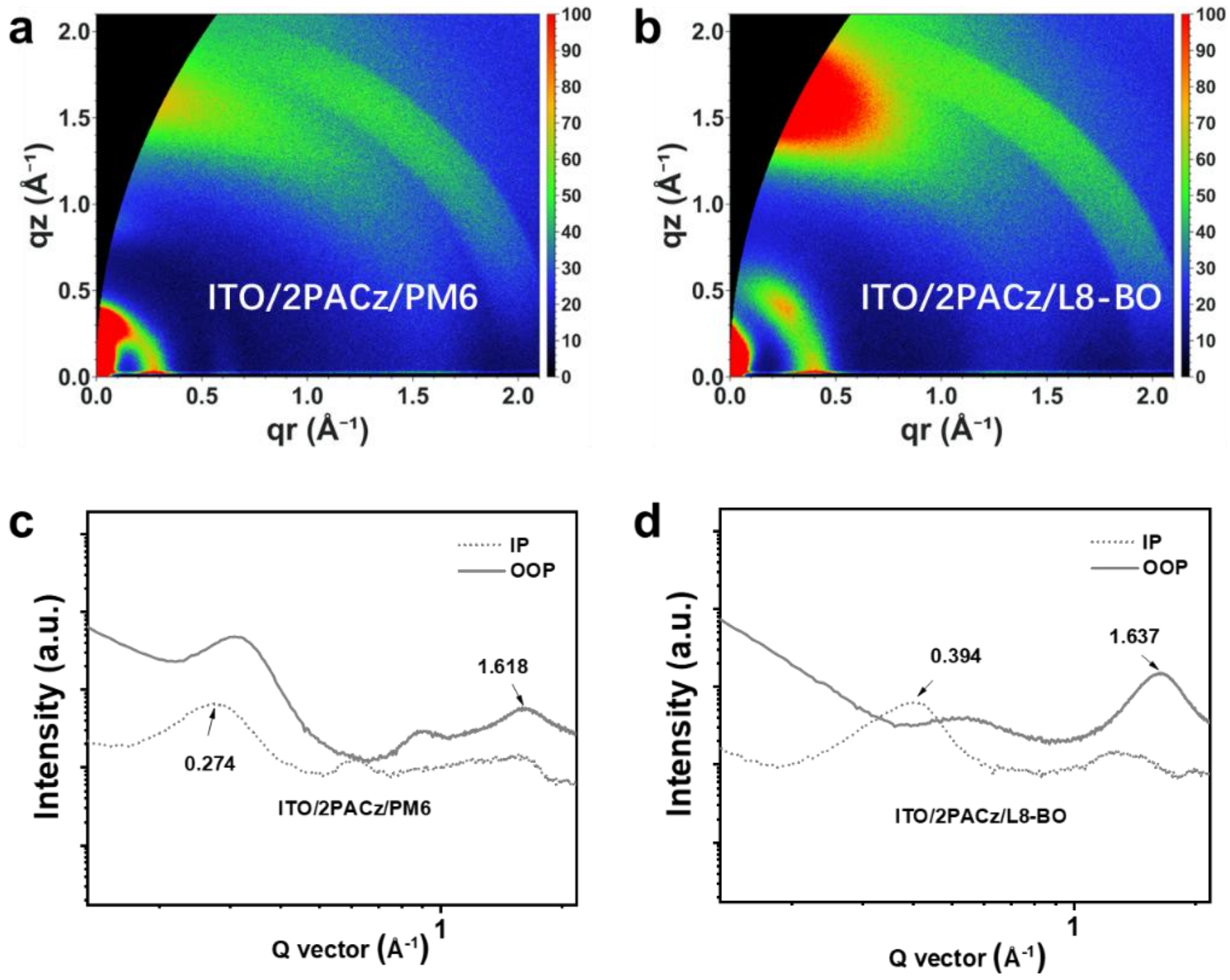

Fig. S23. a-b 2D GIWAXS patterns of donor PM6 and acceptor L8-BO on 2PACz-modified ITO glass substrates, and **c-d** corresponding 1D line cuts.

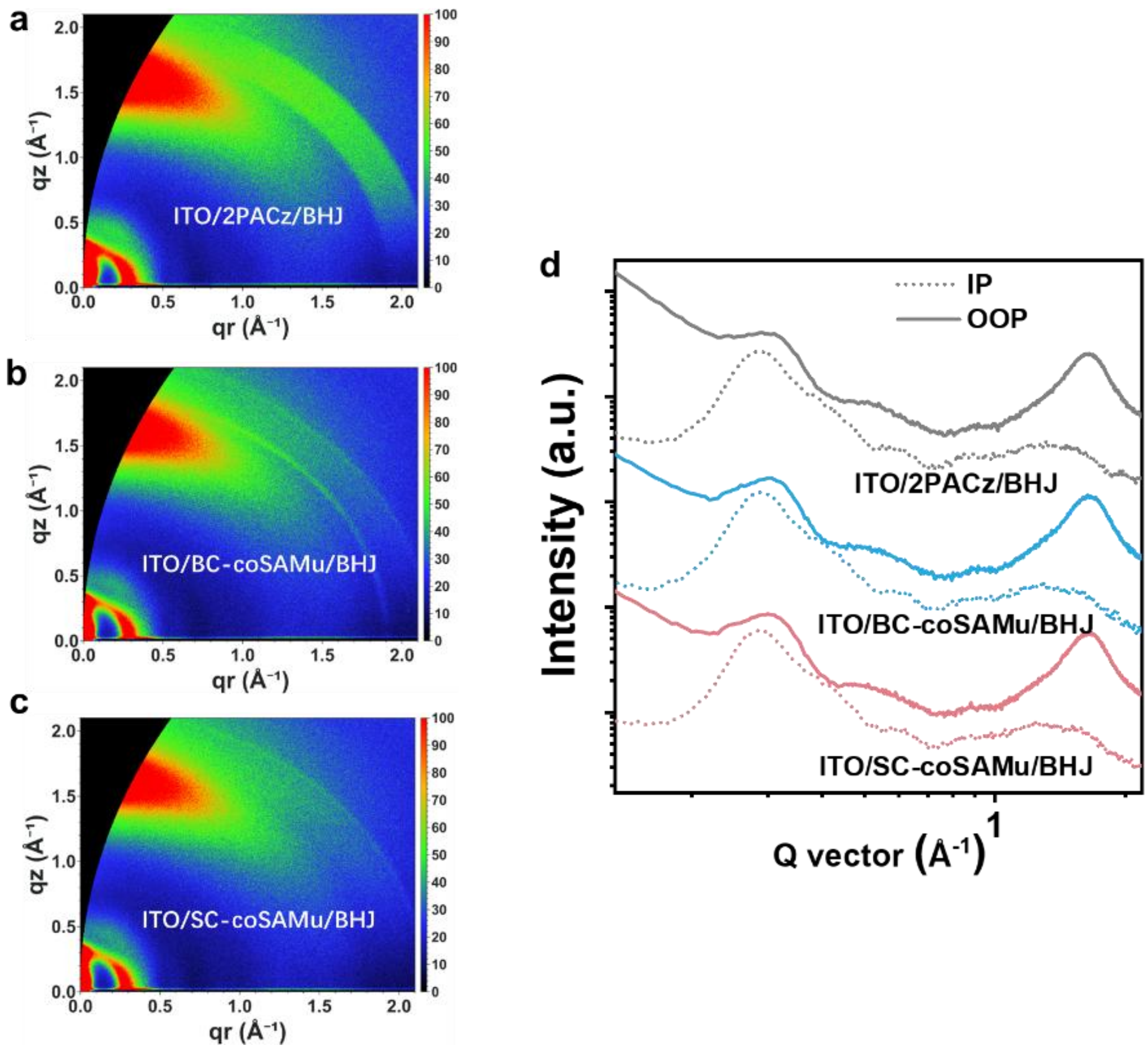

Fig. S24. **a-b** 2D GIWAXS patterns and **d** corresponding 1D line cuts from 2D GIWAXS patterns of PM6:L8-BO bulk heterojunctions formed on 2PACz-, BC-coSAMu-, and SC-coSAMu-modified ITO/glass substrates.

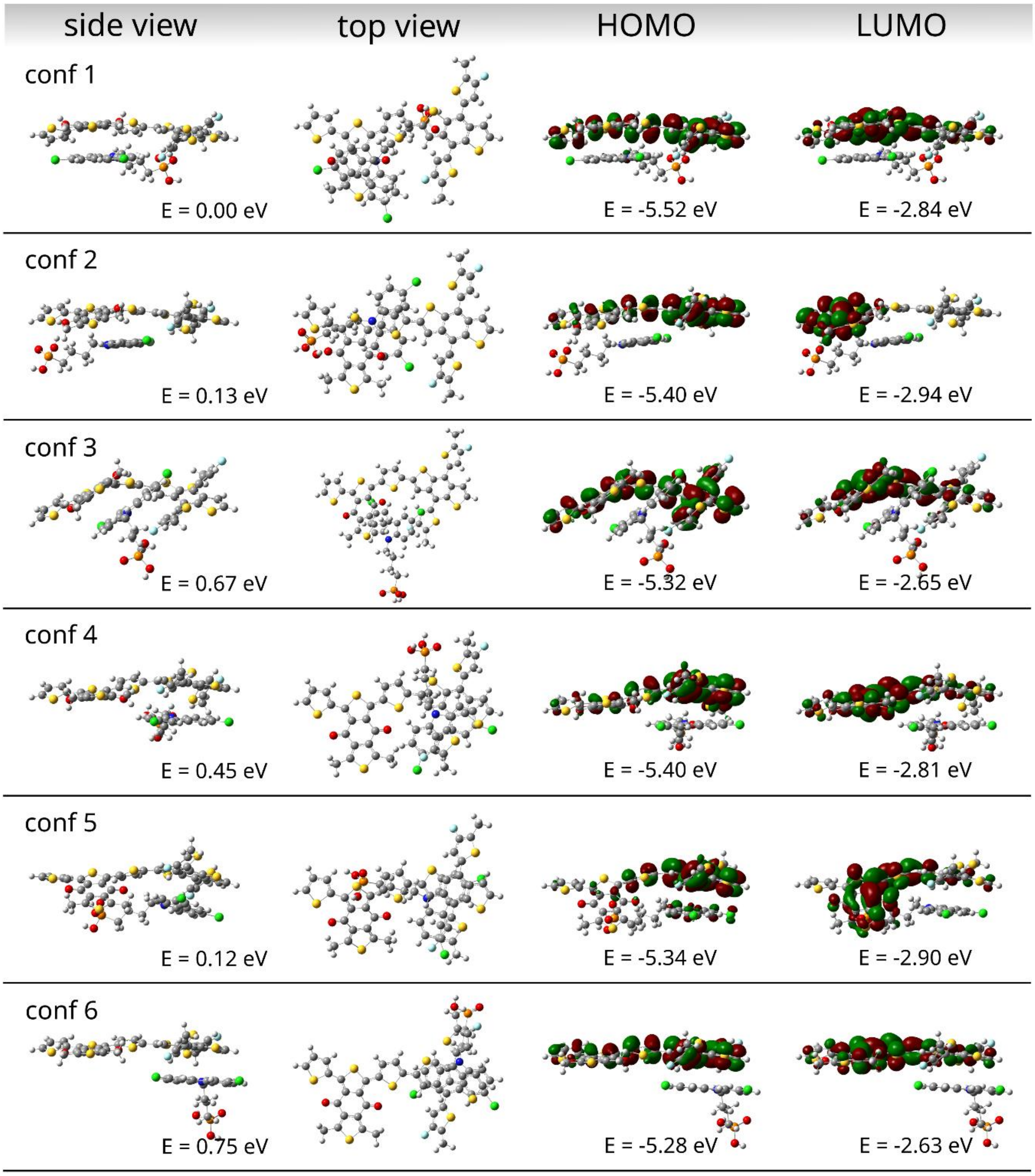

Fig. S25. (left) Side and top views of the optimized PM6/2Cl-4PACz interface geometries, with total electronic energies referenced to the most stable configuration (conf 1). (right) Electronic density distribution and energy levels of the frontier molecular orbitals.

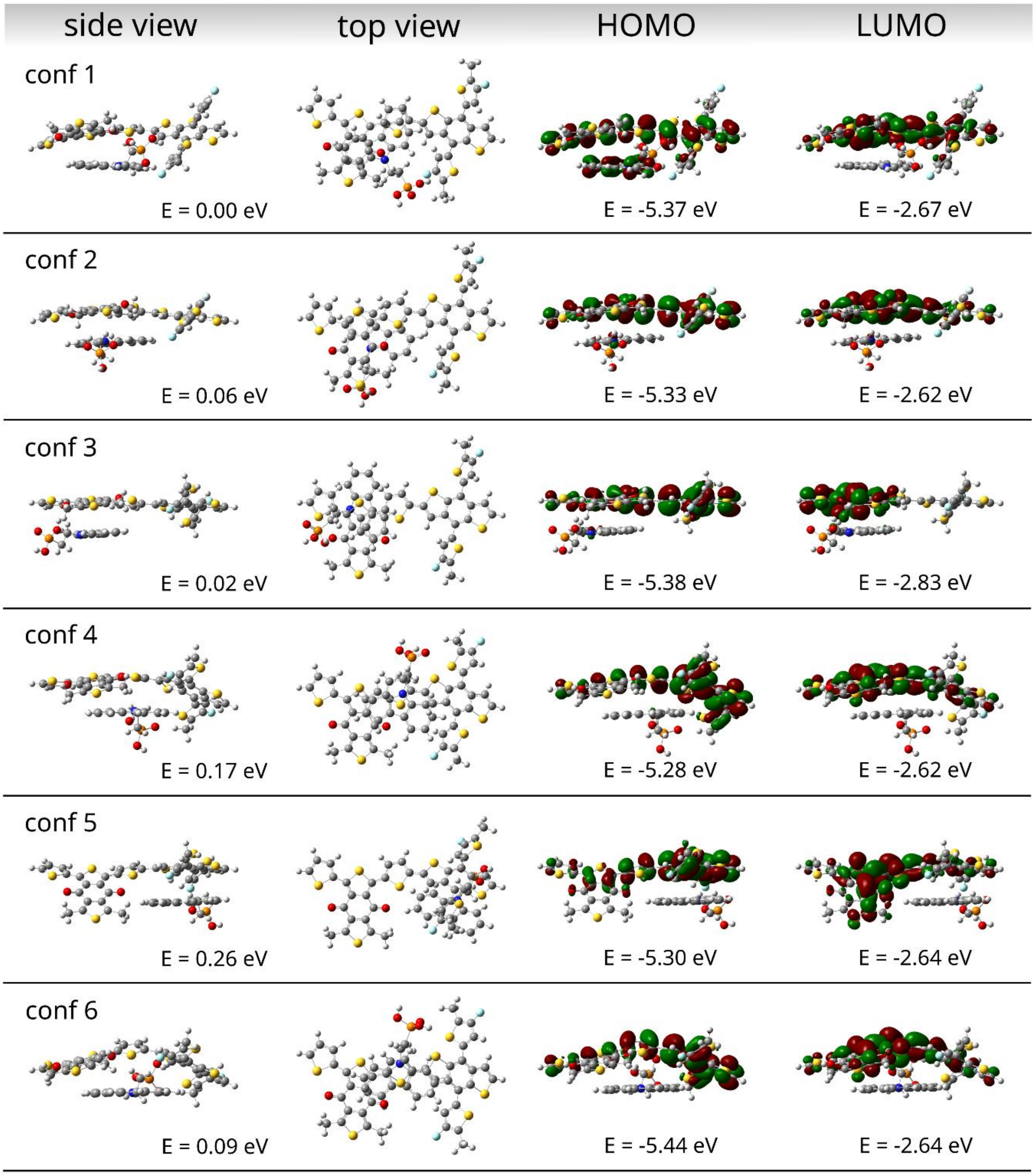

Fig. S26. (left) Side and top views of the optimized PM6/2PACz interface geometries, with total electronic energies referenced to the most stable configuration (conf 1). (right) Electronic density distribution and energy levels of the frontier molecular orbitals.

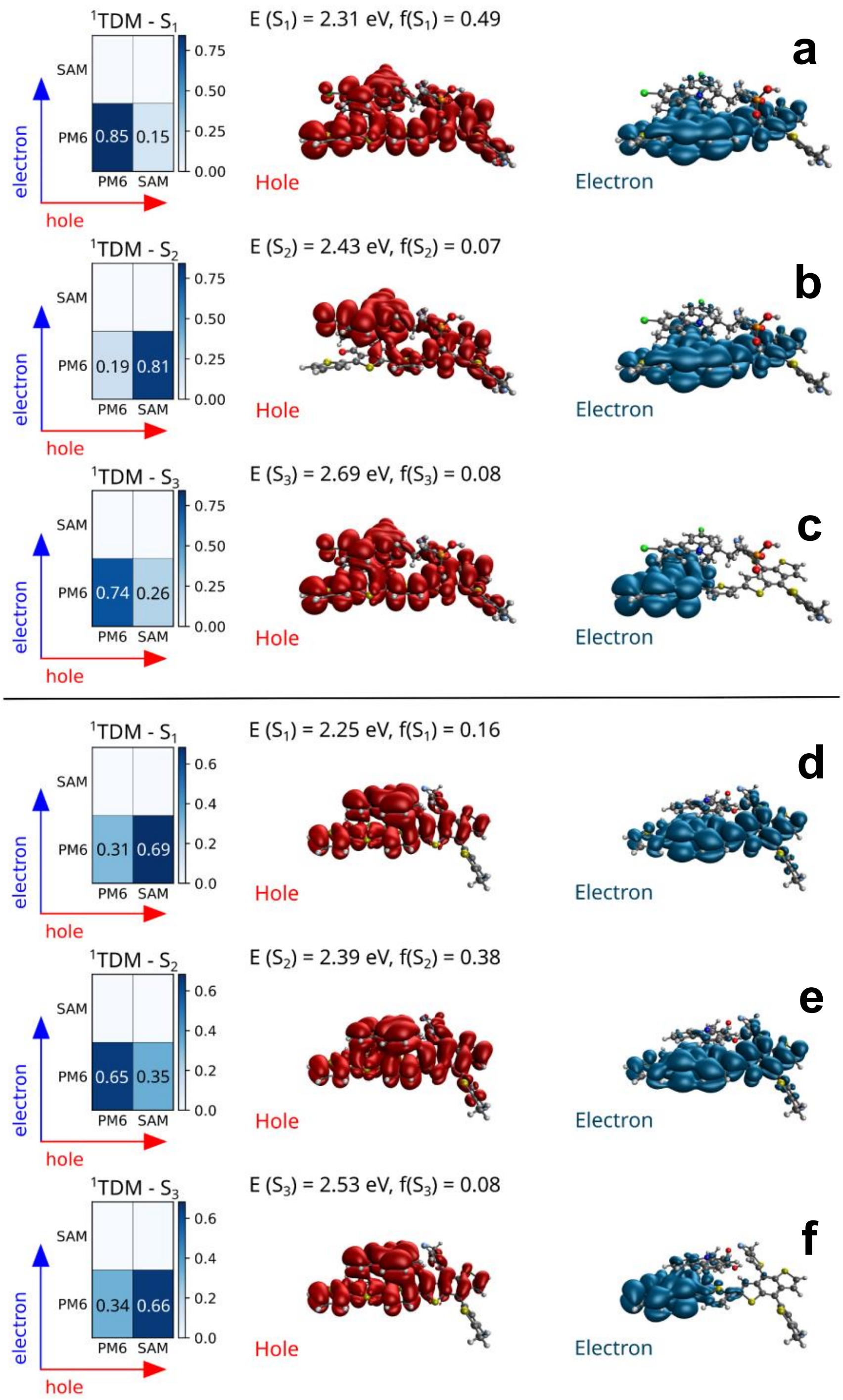

Fig. S27. Excited State Properties of PM6/2Cl-4PACz and PM6/2PACz Interfaces. Panels **a–c** show PM6/2Cl-4PACz and panels **d–f** show PM6/2PACz, each using the most stable configuration (conf 1). The properties of the first three low-lying excited states (S_1, S_2, S_3) are presented. The one-electron transition density matrix (^{1}TDM) characterizes the nature of each excitation as either local or charge-transfer: off-diagonal elements quantify the electron density transferred between PM6 and the SAM, while diagonal elements indicate the local excitation character within PM6 or SAM.

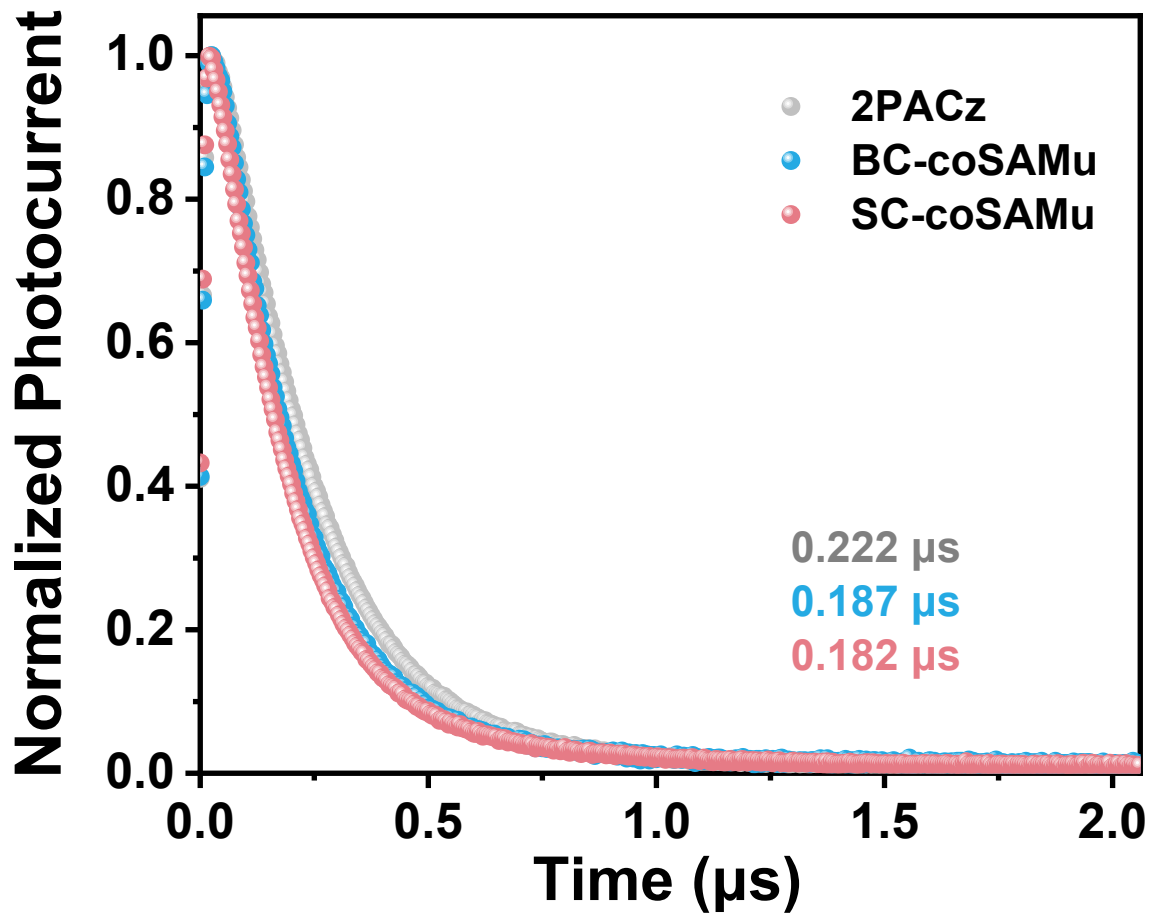

Fig. S28. Transient photocurrent (TPC) response for PM6:L8-BO devices with different HTLs.

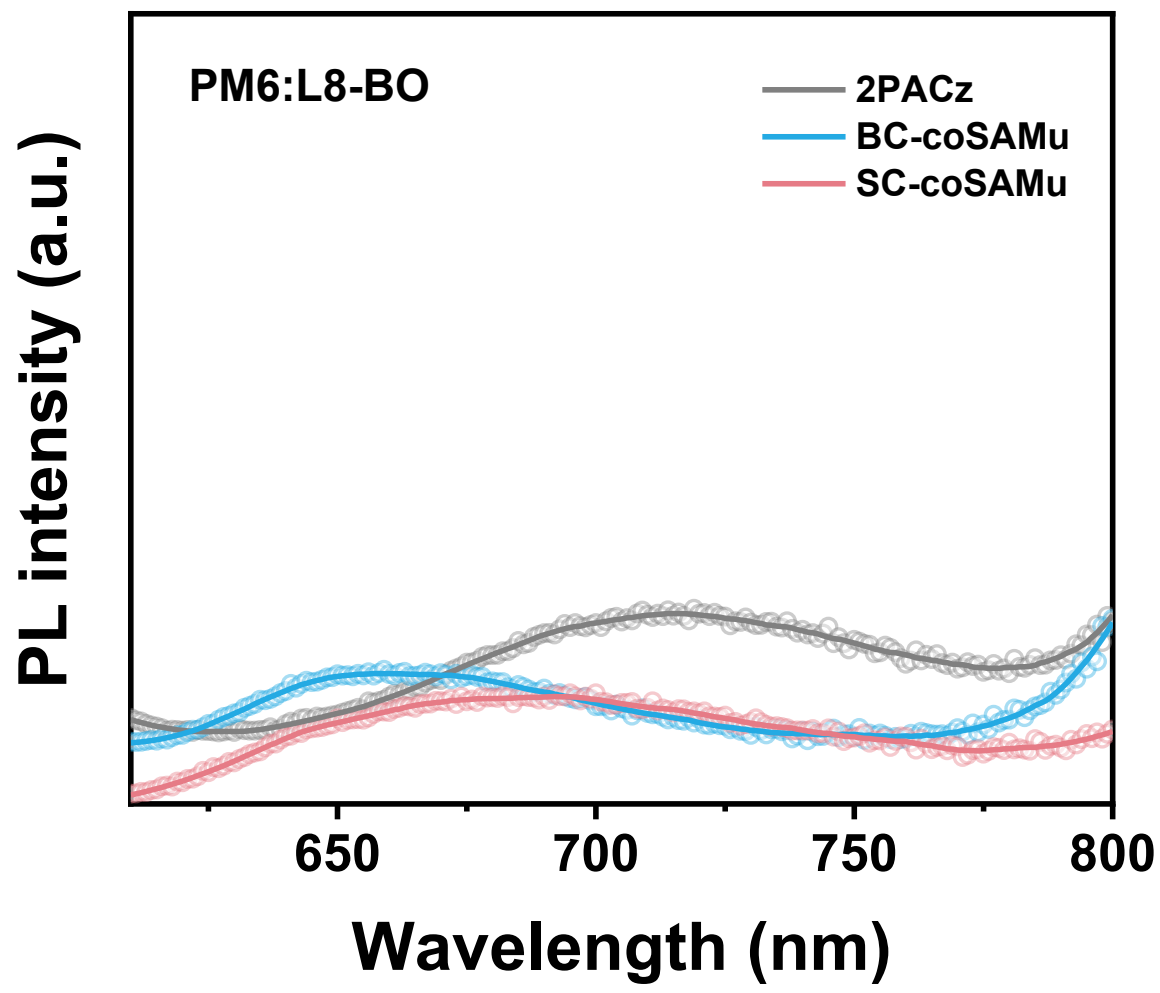

Fig. S29. PL emission of PM6:L8-BO on 2PACz, BC-coSAMu, and SC-coSAMu HTL under 450 nm excitation.

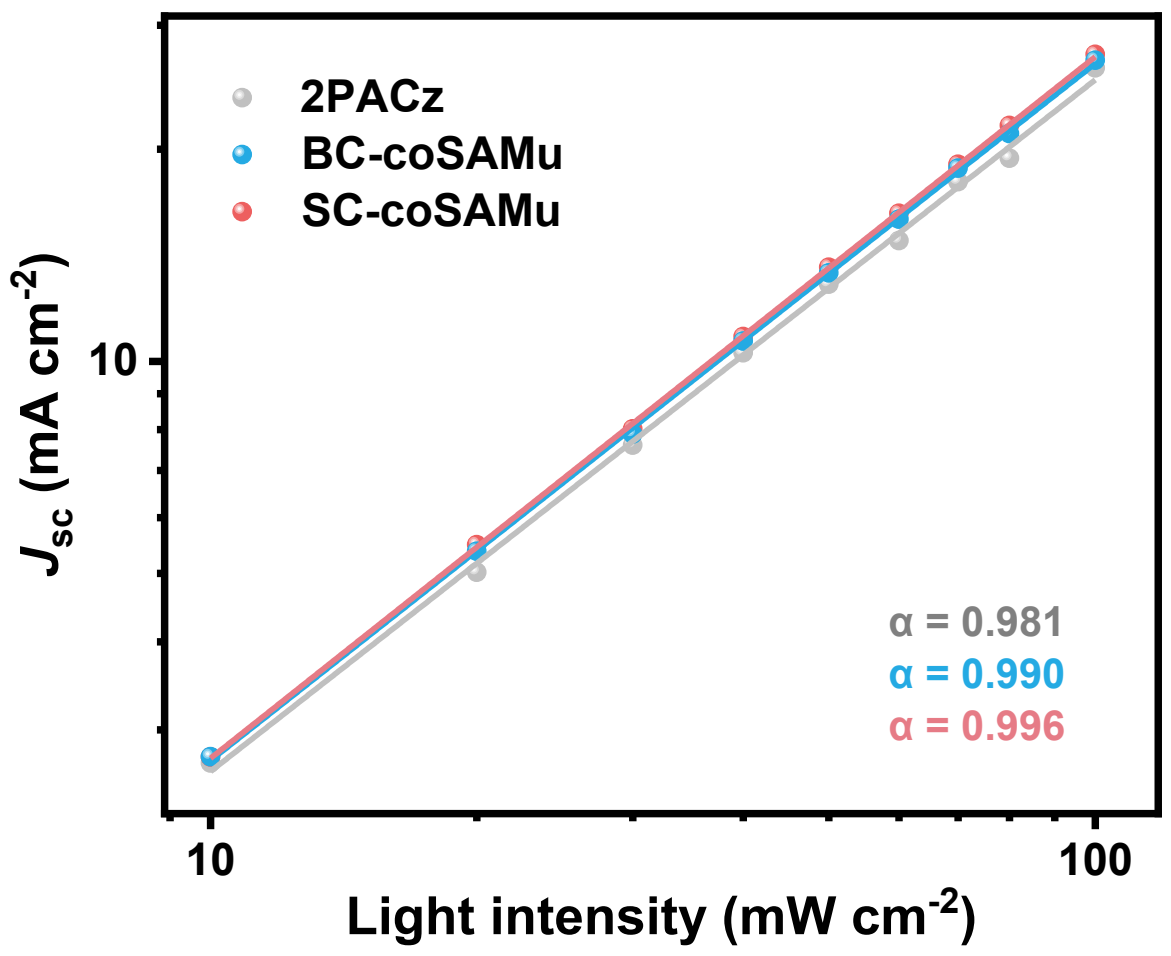

Fig. S30. Light intensity dependence of the *J*sc in PM6:L8-BO devices with different HTLs.

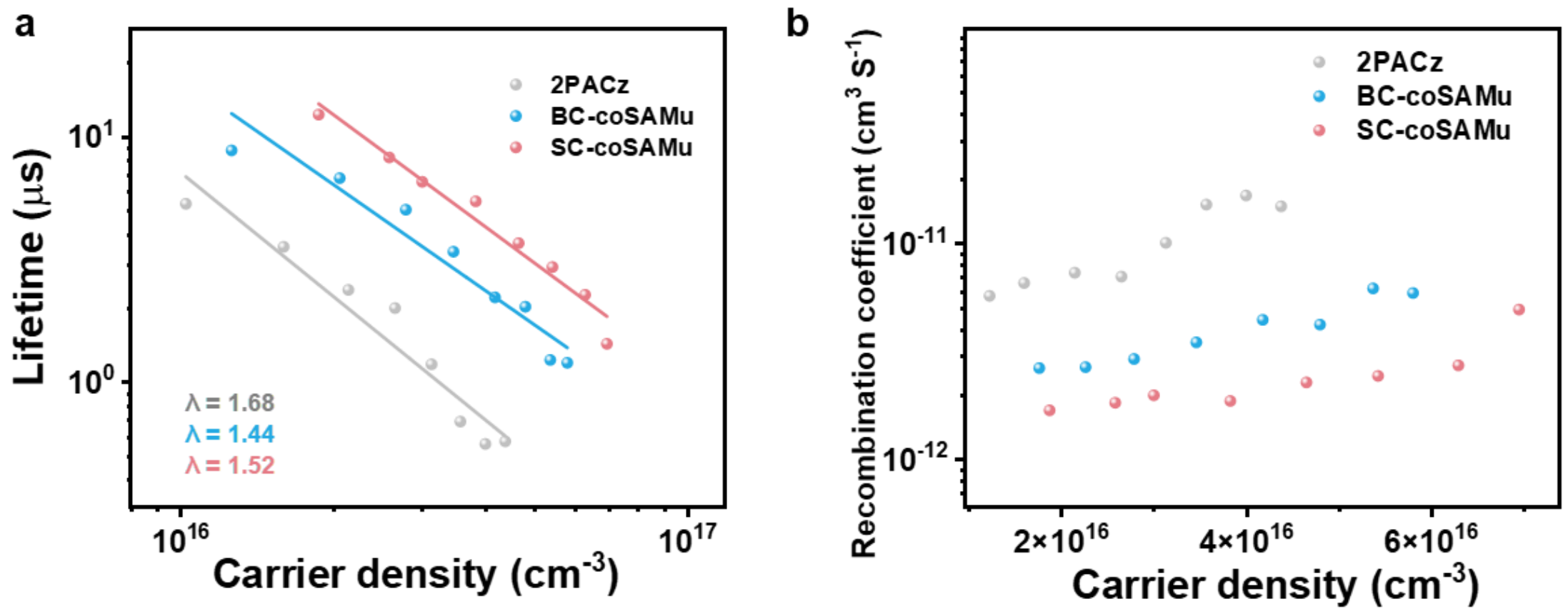

Fig. S31. **a** Carrier lifetime as a function of carrier density, and **b** Bimolecular recombination rate constant (krec) versus carrier density for PM6:L8-BO devices incorporating 2PACz, BC- and SC-coSAMu HTLs, respectively.

S3 Supporting Tables

Table S1. Photovoltaic performance parameters of the OSCs based on PM6:L8-BO with different BC-coSAMu (2PACz:2Cl-4PACz = 1:X, w/w) as HTLs under the irradiation of AM 1.5G, 100 mW/cm^2.

X	V_{oc} [V]	J_{sc} [mA/cm^2]	FF [%]	PCE [%]
0.6	0.892	23.98	73.46	15.7
0.5	0.891	23.09	70.88	14.6
0.4	0.894	24.39	74.35	16.2
0.3	0.891	26.14	81.33	19.0
0.2	0.897	26.27	78.73	18.7
0.1	0.898	26.85	76.81	18.5
0	0.887	25.73	79.66	18.2

Table S2. Mulliken population analysis for groups of atoms of 2PACz and 2Cl-4PACz.

Group or Atoms	2PACz	2Cl-4PACz
Carbazole	-0.19	-0.22
2H or 2Cl (of Carbazole)	0.18	-0.16
N (of Carbazole)	-0.53	-0.54
Alkyl Chain	0.09	0.12
PO_3H_2	0.10	0.10

Table S3. C 1*s*, O 1*s*, N 1*s*, P 2*p*, Cl 2*p* and In 3$d_{3/2}$ core-level peak area as measured by XPS for different SAMus-modified ITO. Relative coverage factor is calculated by normalizing to the In 3$d_{3/2}$ core-level area.

	2PACz		BC-coSAMu		SC-coSAMu	
Core-level	Peak Area/eV	RCF[a)]	Peak Area/eV	RCF[a)]	Peak Area/eV	RCF[a)]
In 3$d_{3/2}$	183432	-	155084	-	154688	-
O 1*s*	57797	0.3151	53598	0.3456	53169	0.3437

C 1*s*	17780	0.0969	17784	0.1147	19200	0.1241
P 2*p*	1562	0.0085	1514	0.0098	1552	0.0100
N 1*s*	2077	0.0113	1811	0.0117	1850	0.0120
Cl 2*p*	-		1192	0.0077	2744	0.0177

a) Relative coverage factors for SAMu-modified ITO surfaces, calculated by normalizing the core-level areas of O 1*s*, C 1*s*, P 1*s*, N 2*p*, Cl 2*p* to the 3$d_{3/2}$ core-level area.

Table S4. C 1*s*, Cl 2*p* and In 3$d_{3/2}$ core-level peak area as measured by XPS for different SAMus-modified ITO. Relative coverage factor is calculated by normalizing to the In 3$d_{3/2}$ core-level area.

ITO/HTL	2PACz (EtOH wash)		BC-coSAMu (EtOH wash)		SC-coSAMu (EtOH wash)	
Core-level	Peak Area/eV	RCF[a)]	Peak Area/eV	RCF[a)]	Peak Area/eV	RCF[a)]
In 3$d_{3/2}$	211981	-	170947	-	219113	-
C 1*s*	13074	0.0617	15739	0.0921	15301	0.0698
Cl 2*p*	-	-	1182	0.0069	882	0.0040

a) Relative coverage factors for SAMu-modified ITO surfaces, calculated by normalizing the core-level areas of C 1*s* and Cl 2*p* to the 3$d_{3/2}$ core-level area.

Table S5. Photovoltaic performance parameters of the OSCs based on PM6:L8-BO with different HTL under the irradiation of AM 1.5G, 100 mW/cm^2.

EtOH washed HTL	V_{oc} [V]	J_{sc} [mA/cm^2]	FF [%]	PCE_{max}[a] [%]
2PACz	0.882	26.23	78.6	18.2 (17.8 ± 0.38)
BC-coSAMu	0.890	26.73	80.2	19.1 (18.7 ± 0.35)
SC-coSAMu	0.888	26.61	80.2	19.0 (18.7 ± 0.30)

[a] The average PCE values in brackets were obtained from 10 devices.

Table S6. Photovoltaic performance parameters of the OSCs based on PM6:L8-BO with different HTLs under the irradiation of AM 1.5G, 100 mW/cm^2.

HTL[a]	V_{oc} [V]	J_{sc} [mA/cm^2]	FF [%]	PCE [%]
A	0.891	26.14	81.3	18.9
B	0.899	26.29	81.5	19.3
C	0.892	23.98	73.5	15.7
D	0.854	23.15	72.3	14.4

[a] A: BC-coSAMu (2PACz:2Cl-4PACz (10:3, w/w));
B: SC-coSAMu (2PACz/2Cl-4PACz);
C: BC-coSAMu (2PACz:2Cl-4PACz (10:6, w/w));
D: SC-coSAMu (2Cl-4PACz/2PACz).

Table S7. Photovoltaic performance parameters of the OSCs based on D18:L8-BO with different HTL under the irradiation of AM 1.5G, 100 mW/cm^2.

HTL	V_{oc} [V]	J_{sc} [mA/cm^2]	FF [%]	PCE_{max} [a] [%]
2PACz	0.901	26.12	79.4	18.4 (18.1 ± 0.25)
BC-coSAMu	0.906	26.81	81.2	19.7 (19.4 ± 0.25)
SC-coSAMu	0.910	27.13	81.4	20.1 (19.8 ± 0.29)

[a] The average PCE values in brackets were obtained from 15 devices.

Table S8. Photovoltaic performance parameters of the OSCs based on PM6:Y6 with different HTL under the irradiation of AM 1.5G, 100 mW/cm^2.

HTL	V_{oc} [V]	J_{sc} [mA/cm^2]	FF [%]	PCE_{max} [a] [%]
2PACz	0.855	26.12	76.3	17.0 (16.8 ± 0.22)
BC-coSAMu	0.857	26.87	78.8	18.1 (18.0 ± 0.13)
SC-coSAMu	0.855	26.72	78.3	17.9 (17.7 ± 0.24)

[a] The average PCE values in brackets were obtained from 15 devices.

Table S9. Photovoltaic performance parameters of the OSCs based on PM6:BTP-eC9 with different HTL under the irradiation of AM 1.5G, 100 mW/cm^2.

HTL	V_{oc} [V]	J_{sc} [mA/cm^2]	FF [%]	PCE$_{max}$ [a] [%]
2PACz	0.853	26.25	78.0	17.5 (17.2±0.31)
BC-coSAMu	0.857	27.18	78.9	18.4 (18.1±0.27)
SC-coSAMu	0.859	27.33	79.0	18.5 (18.3±0.25)

[a] The average PCE values in brackets were obtained from 15 devices.

Table S10. Photovoltaic performance parameters of the OSCs based on PM6:L8-BO with different BC-coSAMu (2PACz:2Cl-3PACz = 1:X, w/w) as HTLs under the irradiation of AM 1.5G, 100 mW/cm^2.

X	V_{oc} [V]	J_{sc} [mA/cm^2]	FF [%]	PCE [%]
0.6	0.879	24.57	71.2	15.4
0.5	0.877	24.73	72.6	15.8
0.4	0.881	25.00	74.1	16.3
0.3	0.882	24.88	75.7	16.6
0.2	0.879	25.04	76.9	16.9
0.1	0.886	25.45	77.1	17.4
0	0.885	25.61	78.7	17.8

Table S11. Photovoltaic performance parameters of the OSCs based on PM6:L8-BO with different BC-coSAMu (2PACz:2Cl-5PACz = 1:X, w/w) as HTLs under the irradiation of AM 1.5G, 100 mW/cm^2.

X	V_{oc} [V]	J_{sc} [mA/cm^2]	FF [%]	PCE [%]
0.6	0.891	26.51	78.0	18.4
0.5	0.893	24.62	78.4	17.2
0.4	0.894	24.42	77.1	16.8
0.3	0.895	25.90	79.9	18.5

0.2	0.895	25.54	77.5	17.7
0.1	0.892	26.05	77.1	17.9
0	0.885	25.61	78.7	17.8

Table S12. Photovoltaic performance parameters of the OSCs based on PM6:L8-BO with different HTL under the irradiation of AM 1.5G, 100 mW/cm^2.

HTL	V_{oc} [V]	J_{sc} [mA/cm^2]	FF [%]	PCE_{max} [%]
2PACz	0.885	25.61	78.7	17.8 (17.6 ± 0.24)[a]
BC-coSAMu-C3	0.882	24.88	75.7	16.6 (16.3 ± 0.35)[a]
SC-coSAMu-C3	0.887	25.23	74.1	16.5 (16.2 ± 0.38)[a]
BC-coSAMu-C4	0.892	26.14	81.3	19.0 (18.7 ± 0.13)[b]
SC-coSAMu-C4	0.899	26.29	81.5	19.3 (19.1 ± 0.15)[b]
BC-coSAMu-C5	0.895	25.90	79.9	18.5 (18.3 ± 0.20)[a]
SC-coSAMu-C5	0.894	26.19	80.7	18.9 (18.8 ± 0.18)[a]

The average PCE values in brackets were obtained from 10 devices [a] and 15 devices [b].

Table S13. Summary of the crystal coherence length (*CCL*) and *d*-spacing of PM6:L8-BO bulk heterojunctions formed on 2PACz-, BC-coSAMu-, and SC-coSAMu-modified ITO/glass substrates.

HTL	LOCATION (Å^{-1})	FWHM (Å^{-1})	*CCL* (Å)	*d*-spacing (Å)
OOP	010	010	010	010
2PACz	1.634	0.32	17.70	3.845
BC-coSAMu	1.640	0.32	17.63	3.832
SC-coSAMu	1.643	0.33	17.15	3.823
IP	100	100	100	100
2PACz	0.289	0.07	83.21	21.709
BC-coSAMu	0.292	0.07	83.61	21.549
SC-coSAMu	0.292	0.07	83.61	21.549

Supplementary References

[1] A. D. Becke, Density-functional thermochemistry. III. The role of exact exchange. J. Chem. Phys. **98**(24), 5648–5652 (1993). https://doi.org/10.1063/1.464913

[2] F. Weigend, R. Ahlrichs, Balanced basis sets of split valence, triple zeta valence and quadruple zeta valence quality for H to Rn: Design and assessment of accuracy. Phys. Chem. Chem. Phys. **7**(18), 3297–3305 (2005). https://doi.org/10.1039/B508541A

[3] M. J. Frisch, G. W. Trucks, H. B. Schlegel, G. E. Scuseria, M. A. Robb, J. R. Cheeseman, G. Scalmani et al. Gaussian 16, Revision C.01. Gaussian, Inc., Wallingford, CT (2016).

[4] F. Plasser, TheoDORE: A toolbox for a detailed and automated analysis of electronic excited state computations. J. Chem. Phys. **152**(8), 084108 (2020). https://doi.org/10.1063/1.5143076

[5] Q. Chen, K. Sun, L. R. Franco, J. Wu, L. Öhrström et al., Effects of alkyl spacer length in carbazole-based self-assembled monolayer materials on molecular conformation and organic solar cell performance. Adv. Sci. **12**(19), 2410277 (2025). https://doi.org/10.1002/advs.202410277

[6] Y. Lin, Y. Firdaus, F. H. Isikgor, M. I. Nugraha, E. Yengel et al., Self-assembled monolayer enables hole transport layer-free organic solar cells with 18% efficiency and improved operational stability. ACS Energy Lett. **5**(9), 2935–2944 (2020). https://doi.org/10.1021/acsenergylett.0c01421

[7] J. P. Perdew, K. Burke, M. Ernzerhof, Generalized Gradient Approximation Made Simple. Phys. Rev. Lett. **77**(18), 3865–3868 (1996). https://doi.org/10.1103/PhysRevLett.77.3865

[8] P. E. Blöchl, Projector Augmented-Wave Method. Phys. Rev. B **50**(24), 17953–17979 (1994). https://doi.org/10.1103/PhysRevB.50.17953

[9] G. Kresse, D. Joubert, From Ultrasoft Pseudopotentials to the Projector Augmented-Wave Method. Phys. Rev. B **59**(3), 1758–1775 (1999). https://doi.org/10.1103/PhysRevB.59.1758

[10] S. Grimme, S. Ehrlich, L. Goerigk, Effect of the damping function in dispersion corrected density functional theory. J. Comput. Chem. **32**(7), 1456–1465 (2011). https://doi.org/10.1002/jcc.21759

[11] G. Kresse, J. Hafner, Ab initio molecular dynamics for open-shell transition metals. Phys. Rev. B **48**(17), 13115–13118 (1993). https://doi.org/10.1103/PhysRevB.48.13115

[12] G. Kresse, J. Furthmüller, Efficient iterative schemes for ab initio total-energy calculations using a plane-wave basis set. Phys. Rev. B **54**(16), 11169–11186 (1996). https://doi.org/10.1103/PhysRevB.54.11169

[13] M. J. Abraham, T. Murtola, R. Schulz, S. Páll, J. C. Smith et al., GROMACS: High performance molecular simulations through multi-level parallelism from laptops to supercomputers. SoftwareX 1–2, 19–25 (2015). https://doi.org/10.1016/j.softx.2015.06.001

[14] W. L. Jorgensen, D. S. Maxwell, J. Tirado-Rives, Development and testing of the OPLS all-atom force field on conformational energetics and properties of organic liquids. J. Am. Chem. Soc. **118**(45), 11225–11236 (1996). https://doi.org/10.1021/ja9621760

[15] W. L. Jorgensen, J. Tirado-Rives, Potential energy functions for atomic-level simulations

of water and organic and biomolecular systems. Proc. Natl. Acad. Sci. U.S.A. **102**(19), 6665–6670 (2005). https://doi.org/10.1073/pnas.0408037102

[16] L. S. Dodda, I. C. de Vaca, J. Tirado-Rives, W. L. Jorgensen, LigParGen web server: an automatic OPLS-AA parameter generator for organic ligands. Nucleic Acids Res. **45**(W1), W331–W336 (2017). https://doi.org/10.1093/nar/gkx312

[17] J. Wu, G. Li, J. Fang, X. Guo, L. Zhu et al., Random terpolymer based on thiophene-thiazolothiazole unit enabling efficient non-fullerene organic solar cells. Nat. Commun. **11**(1), 4612 (2020). https://doi.org/10.1038/s41467-020-18378-9
